# Response of soil bacteria on habitat-specialization and abundance gradient to different afforestation types

**DOI:** 10.1038/s41598-023-44468-x

**Published:** 2023-10-24

**Authors:** Zhenlu Qiu, Jie Li, Peng Wang, Dong Wang, Li Han, Xiaojuan Gao, Jing Shu

**Affiliations:** 1 College of Forestry Engineering, Shandong Agricultural and Engineering University, Jinan, 250100 China; 2 College of Biological and Chemical Enginering, Qilu Institute of Technology, Jinan, 250200 China

**Keywords:** Ecology, Microbiology, Ecology

## Abstract

Studies involving response of subgroups of soil microorganisms to forest change, especially comparative studies on habitat-specialization and abundance gradient were still lack. In this study, we analyzed the response of soil bacterial diversity and structure to afforestation types and its relationship to environment of Fanggan ecological restoration area under the classification of subgroups by habitat-specialization and abundance gradient based on abundance ratio respectively. The results were: (1) On the habitat-specialization gradient, the variation of OTUs species number and abundance was consistent and positively correlated with habitat-specialization; on the abundance gradient, the variation was opposite and OTUs species number was negatively correlated with abundance gradient; (2) The distribution frequency of each subgroup on both gradients was the highest in broad-leaved forests, but the abundance was the opposite. The distribution frequency of the same stand showed no difference among habitat-specialization subgroups, but the abundant subgroup in broad-leaved forests was the highest among the abundance subgroups; (3) α-diversity was positively correlated with habitat-specialization but negatively with abundance, with the highest mostly in broad-leaved and mixed forests; (4) Community structure among stands on habitat-specialization gradient showed no significant difference, but that of rare subgroup between broad-leaved forests and other stands significantly differed. Plant diversity and vegetation composition correlated stronger with community structure than spatial distance and soil physicochemical properties on both gradients. Our results provided a new perspective for revealing the effects of afforestation types on soil bacteria from the comparison of habitat specialization and abundance gradient.

## Introduction

Forest ecosystems played a crucial role in generating primary productivity, regulating nutrient biogeochemical cycles and providing a variety of ecological services^1^. But with the rapid development of economy and society, a large number of natural forests have been deforested to meet the demand for wood, which has been seriously damaged the forest ecosystems^2,3^. According to historical records, the primary forests in Shandong were almost cut down in the Qin and Han dynasties, and the secondary forests were also destroyed repeatedly. Therefore, many areas of Shandong province had been bare rocks, and some had been artificial or secondary forests^4^. Recent studies have shown that the barren hills in the northern Shandong central mountain area of began to suffering increasing forest cutting and land reclamation activities in the fifteenth century^5^. During the sixteenth century to the eighteenth century, the local virgin forests were seriously destroyed or even cleared away, left barren mountains^5^. To alleviate the crisis of the deforestation of natural forest, plantations have been rapidly rised and related researches have attracted the attention of researchers. However, plantations in Shandong central mountain area began late, in the 1960s. Due to poor soil nutrients, conifers such as *Platycladus orientalis* and *Pinus tabulaeformis* were mainly planted^6^.

Soil microorganisms are key components that link the nutrient composition and cycle between above and below ground of forest ecosystem^7,8^. In recent years, interests in the factors that shape and control soil microbial community composition and diversity have been spring up^9^, especially since the rise of high-throughput sequencing. Factors such as vegetation type, soil physicochemical properties, plant diversity and spatial distance could affect the composition and structure of soil microbial community^10–12^. These factors interacted with each other, but their relative importance to soil microbial diversity and community structure remains to be explored. Vegetation type was likely to be the main factor shaping soil microbial diversity and community structure^13,14^. Different vegetation types affect soil microbial diversity and community structure through altering the chemical composition and content of litter and root exudates, making them strongly related^15,16^. Vegetation types might also affect microbial community structure by regulating soil physicochemical properties^17^, in which soil pH, organic carbon could directly affect soil microbial community composition^18^. Soil pH, carbon nitrogen ratio, etc., have also been shown vital for soil microbial community structures^19,20^. Studies have shown large differences in soil microbial community structure between coniferous forests and broad-leaved forests^21^. In addition, high plant diversity contributed to the accumulation of soil organic matter and the preservation of soil biodiversity^22–24^. Studies have also shown that diffusion being the main reason for the differences in soil microbial community structure^25,26^, this was often the case in nutrient adequate ecosystems, because the impact of space distance on community structure was positively correlated with nutrient adequacy^27^. Relevant studies have mainly focused on tropical forests^22,28^, subtropical forests^23,29^, temperate forests^30^and shrubs^13^, neglected the effect of afforestation types on soil microbial diversity and community structure of barren hills in the north warm temperate zone.

Although many factors have been proved to affect soil microbial diversity and community composition, different taxa responded different to environmental factors. For example, fungal diversity was more sensitive to stand changes than bacteria^31^, while the latter was more sensitive to soil physicochemical properties^32,33^. Different abundance and different habitat-specialization subgroups differed in their ability to adapt to and coexist with the environment, and their responses to soil environmental changes were also different. Abundant subgroup tended to include fewer species and were widely distributed, and they tended to dominate community functions^34^. Rare subgroup tended to include more species and more specialized distribution^35,36^. Previous studies have more focused to abundant subgroup which dominated community functions, because they often played a key role in organic matter flow and biomass accumulation^26^, while rare subgroup has been ignored for a long time. However, the latter also played a key role in maintaining biodiversity and promoting nutrient cycling and the function of diverse microbial communities^37,38^. On the abundance gradient, we defined the permanent abundant subgroup (relative abundance > 1% in all quadrats) and the conditional abundant subgroup (relative abundance > 0.01% in all quadrats and > 1% in some quadrats) as abundant subgroup, the permanent rare subgroup (relative abundance < 1% in all quadrats) and conditional rare subgroup (relative abundance < 0.01% in some quadrats and less than 1% in all quadrats) as rare subgroup and the rest were defined as medium abundance subgroup^39^. On the gradient of habitat-specialization, the subgroup distributed evenly in each resource environment were classified into habitat-generalized subgroup. The poor niche differentiation and severe uneven distribution caused by habitat changes were classified into habitat-specialized subgroup. The rest were habitat-neutral subgroup^40^. The classification of habitat-specialization was based on the comparison between the actual niche width of OTUs and the confidence interval of zero niche width model. Those above the limit of 95% confidence interval was classified into habitat-generalized subgroup, and those below the limit of 5% confidence interval was divided into habitat-specialized groups, and those between limit of 5 and 95% confidence interval was divided into habitat-neutral groups^41^. In this study, the responses of soil bacteria to different afforestation types were studied in a comparative way at both habitat-specialization and abundance gradients, so as to reveal the differences and relationships of the above responses between abundance and niche differentiation.

In the Fanggan ecological restoration area located in the northern part of Shandong Central Mountain area, although there were plantations before the 1970s, the growth and forest formation effect was little, and the restoration area was mostly Barren mountain and shrub (s) vegetation landform. Over the past 50 years, local villagers have moved 20,000,000 m^3^ of earth and planted more than 3,000,000 trees with different afforestation types, including coniferous forests (CF), mixed coniferous and broad-leaved forests (MF) and broad-leaved forests (BF). So far, the ecological restoration area has formed the forest ecosystem with the coverage rate of more than 90% and a complete vertical community structure of tree, shrub and grass. At present, the restoration area includes dense forest canopy zone, gaps with sparse forest canopy zone, dense shrub zone and bare rock lacking vegetation zone. The initial soil conditions of the restoration area were consistent, so the current soil properties and microbial changes could reflect the effects of different afforestation types on soil characteristics. The results could not only reveal the differences and mechanisms of response of soil bacterial to different afforestation types, but also provide valuable reference and guidance for Fanggan village to further strengthen and adjust vegetation restoration strategies.

## Materials and methods

### Study site

Fanggan ecological restoration area, locate in the northern part of Shandong Central Mountain area (117°24′45″–117°28′5″E, 36°24′23″–36°26′44″N), was mountainous and hilly terrain with the highest altitude 860 m and more than 30 hilltops above 400 m. The climate type of the restoration area is continental monsoon climate with distinct features of four seasons and the same period of rain and heat. The average annual temperature was 12.4 °C, the lowest extreme temperature was − 22.5 °C and the average annual precipitation was more than 830 mm, concentrate in July to September. The soil type in the restoration area was mountain brown soil, and the average thickness of litter was 4 cm. The vertical structure of vegetation community was obvious, but the species were relatively simple. The tree layer mainly included *Pinus densiflora*, *P. tabulaeformis*, *Populus davidiana*, *Robinia pseudoacacia* and *Diospyros lotus*, the shrub layer was mainly consisted of *Vitex negundo* var. *heterophyll*, *Ziziphus jujuba* var. *spinosa*, *Grewia biloba* and the regeneration seedlings of the tree layer, the herbaceous layer was consisted mainly of some species of Gramineae, Cyperaceae and Compositae. The plantations in the restoration area were all planted between 1975 and 1985, with relatively consistent forest age.

### Vegetation survey and soil sampling

Through literature review and field investigation, forests of the restoration were mainly distributed in the altitude range of 300–750 m, so the vegetation community investigation was carried out in dense forest canopy region of the altitude range. Because the original planting area of different forest types was quite different, this study allocated the quadrat quantity according to the actual area proportion of each forest type. A total of 20 quadrats including 17 quadrats of 3 forest types of plantations, namely, 3 coniferous forests (CF), 3 coniferous broad-leaved mixed forests (MF) and 11 broadleaved forests (BF) as well as 3 shrubs quadrats as comparison were set up. In order to reduce the influence of topographic factors, each quadrat contained a consistent amplitude of topographic variation as far as possible, and the geographical coordinates, elevation, slope direction and slope have been recorded (Table 1). The area of tree layer quadrat was 10 m × 10 m, individuals with DBH ≥ 3 cm were measured per tree, and the species name, coverage, quantity, base diameter and height of shrubs as well as species name, height and coverage of herbs were also recorded. The survey area of each shrub quadrats was 5 m × 5 m, and the statistics of herb layer were carried out. The α-diversity indexes calculated of vegetation community includes Shannon-winner index (Eq. 1), Simpson diversity index (Eq. 2), Shannon evenness index (Eq. 3), Simpson evenness index (Eq. 4), Pielou evenness index (Eq. 5) and species richness. 0–10 cm topsoil and 20–30 cm subsoil were collected in each quadrat according to the five-point sampling method with sampling points arranged at the center and four corners (3 m from the quadrat’s vertex) of the quadrat^35,42^. The samples from same layer at 5 points in each quadrat were thoroughly mixed and divided into two portions. So, there were totally 80 soil sample, for one part was stored in liquid nitrogen for molecular biology extraction and analysis, and the other was stored at room temperature and brought back to the laboratory for physicochemical analysis as soon as possible.

1 $${\text{Shannon-Winner}}\;{\text{diversity}}\;{\text{index}} = - \mathop \sum \limits_{i = 1}^{m} P_{i} \ln P_{i}$$

2 $${\text{Simpson}}\;{\text{diversity}}\;{\text{index}} = 1 - \mathop \sum \limits_{i = 1}^{m} \frac{{P_{i} \left( {P_{i} - 1} \right)}}{{N\left( {N - 1} \right)}}$$

3 $${\text{Shannon}}\;{\text{evenness}}\;{\text{index}} = \frac{Shannon - winner\;diversity\;index}{{{\text{log}}\left( m \right)}}$$

4 $${\text{Simpson}}\;{\text{evenness}}\;{\text{index}} = \frac{Simpson\;diversity\;index}{{{\text{log}}\left( m \right)}}$$

5 $${\text{Pielou}}\;{\text{evenness}}\;{\text{index}} = \frac{{ - \mathop \sum \nolimits_{i = 1}^{m} P_{i} \log P_{i} }}{{{\text{log}}\left( m \right)}}$$

**Table 1:** Community information of shrub and afforestation forests.

Quadrat	Elevation (m)	Longitude (°)	Latitude (°)	Slope (°)	Aspect (°)	Coverage (%)	Dominant species
Shrub (S)	400	117.55062	36.45683	6.2	S189	65	*Vitex negundo* var. *heterophylla*
	381	117.43693	36.40997	21.1	SE131	95	*V. negundo* var. *heterophylla*
	301	117.56891	36.48426	2.3	W270	70	*V. negundo* var. *heterophylla*
Coniferous Forests (CF)	758	117.42622	36.42059	28.7	E81	45	*Pinus. tabuliformis, P.densiflora*
	484	117.45266	36.45675	16.7	W269	60	*P. tabuliformis, P.densiflora*
	458	117.56334	36.42401	38.8	NE47	60	*P. tabuliformis, P.densiflora*
Mixed Forests (MF)	526	117.44411	36.42148	13.5	NW303	60	*P.densiflora, Populus davidiana*
	469	117.45251	36.45733	19.8	W256	40	*Pinus densiflora, Quercus acutissima*
	448	117.45253	36.45747	22.8	N18	70	*P.densiflora, Robinia pseudoacacia*
Broad-leaved Forests (BF)	542	117.44382	36.42024	16	W248	80	*R. pseudoacacia*
	540	117.43137	36.42351	15.8	NE28	55	*Q. acutissima, Diospyros lotus,* *Salix matsudana*
	447	117.48876	36.42349	2.5	N338	70	*Populus davidiana*
	396	117.44651	36.44243	8.2	E69	85	*R. pseudoacacia*
	390	117.44973	36.456	1.4	NW327	85	*P. davidiana*
	390	117.43558	36.41352	2.5	S170	65	*Pterocarya stenoptera,* *D. lotus,* *Amygdalus persica*
	388	117.43705	36.41067	25.4	E82	95	*R. pseudoacacia,* *Koelreuteria paniculata*
	380	117.46176	36.42259	22.3	E111	75	*R. pseudoacacia*
	339	117.44869	36.4427	3.3	S167	60	*R. Pseudoacacia, Q. acutissima, Platycladus orientalis*
	324	117.44886	36.44234	6.4	SE142	80	*Q. acutissima*
	317	117.4665	36.46934	12.7	NW297	70	*P. davidiana*

In the above equations, *m* represents the number of species in the quadrat, *N* represents the total number of individuals of all species, and *Pi* represents the important value of species i in the quadrat. The diversity index of a stand is equal to the average value of the index for the various sides of the stand.

### Determination of soil physicochemical properties

Soil moisture and dry matter content were determined by gravimetric method^43^, pH was determined by potentiometric method using 2.5:1 water-soil ratio. Organic carbon content was determined by potassium dichromate oxidation-spectrophotometry^44^, and available phosphorus was determined by molybdenum-antimony anti-color spectrophotometry^45^. We used potassium chloride solution extraction-spectrophotometry and ultraviolet spectropotometry to determine ammonium nitrogen (NH_4_^+^-N) and nitrate nitrogen (NO_3_^−^-N) respectively. The content of nitrite nitrogen (NO_2_^−^-N) was determined by naphthalene ethylenediamine hydrochloride color spectrophotometry^46,47^. The model of the spectrophotometer is T6 UV–visible spectrophotometer produced by Beijing Puxi General Instrument Co., LTD. The pH meter model is SEvenExcellence S400-Basic made by Mettler Toledo Corporation.

### High throughput sequencing of soil microorganisms

Soil bacterial DNA was extracted by TGuide S96 magnetic bead method and 16S rDNA fragments of DNA were amplified by primers 515F (50-GTGYCAGCMGCCGCGGTAA-3) and 926R (50 -CCGYCAATTYMTTTRAGTTT-30). The PCR products were purified by the VAHTS-TM DNA Clean Beads method and quantified. Unique barcode was used to separate each sample to prevent cross-contamination. Sequencing and data processing (1) Filter of raw data quality Trimmomatic^48^ was used to filter dual-end sequencing files. Parameter setting: Window size was set as 50 bp. The reads will be cut from the start of the window once average Q-score within the window is lower than 20. (2) Identification and removal of primer sequences Cutadapt (Version 1.9.1) was used to identify primer sequences according to the parameters allowing the maximum error ratio of 20% and the minimum coverage of 80%^49^. (3) Double-ended reads splicing USEARCH (Version 10) was used for sample double-ends reads stitching according to the minimum overlap length of 10 bp, the minimum similarity of 90% allowed in overlap area and maximum error base number of 5 bp^50^. (4) Removal of chimera The criteria of chimera are as follows: divide query sequence into chunks without overlap and compare them with the database; The best match of each chunk in the database is selected, and the two best parent sequences are finally selected. The sequence to be detected was compared with the two parents. If a sequence of the two parents has greater than 80% similarity to the Query sequence, the query was judged to be a chimera. Use UCHIME (Version 8.1) to remove chimeras^51^. (5) OTUs clustering and species annotation Cluster analysis was performed using UPARSE at the 97% similarity level, and the usearch command was used to remove affine sequences and singleton OTUs in the process. Each clustered OTUs was annotated into seven subgroupomic classes of boundary, phylum, order, family, genus and species by the sine method through the 16 s in silva or the ITS database in Unite. The community abundance tables for each rank were obtained. The present study was carried out mainly on the OTUs subgroupomic level and the following analysis were all based on the OTUs abundance table.

### Data analysis

#### Classification on abundance gradient

In this study, to estimate the relative abundance of abundant and rare subgroups the thresholds were defined as 1 and 0.01% of the total sequence reads^52,53^. Further to subdivide bacterial communities, the six categories were defined with reference to recent publications^54,55^: (1) permanent abundant subgroup (AAT): relative abundance great than 1% in all samples, (2) permanent rare subgroup (ART): relative abundance less than 0.01% in all samples, (3) conditional medium abundance subgroup (MT): relative abundance between 0.01 and 1% in all samples, (4) conditionally abundant subgroup (CAT): relative abundance great than 0.01% in all samples and 1% in some samples, (5) conditionally rare subgroup (CRT): relative abundance less than 0.01% in some samples but never great than 1% in any sample, (6) conditionally rare and abundant subgroup (CRAT): relative abundance ranging from 0.01% to 1%. The above AAT and CAT were classified as abundant subgroup, ART and CRT as rare subgroup, and MT and CRAT as medium abundance subgroup.

#### Classification on habitat-specialization gradients

Levins niche width (Eq. 6) was calculated using the ‘spaa’ package of R v 4.0.0^56^. The frequency of occurrence of each OTU was randomized and rearranged 1000 times using the replacement method of the ‘EcolUtilis’ package, respectively. Then the zero distribution of the niche width index was calculated for each OTU. Based on the consensus that habitat-generalized subgroup owe a wider niche width than the habitat-specialized subgroup^40^, OTUs were classified into a habitat-generalized or a specialized group based on whether the frequency of observed occurrence exceeded the upper 95% confidence interval or fell below the lower 5% confidence interval, and the OTUs were distributed to the habitat-neutral group if the observed niche width was within in the 5% to 95% confidence interval range^18^.

6 $${\mathbf{B}}_{{\mathbf{i}}} = \frac{1}{{\mathop \sum \nolimits_{{{\varvec{j}} = 1}}^{{\varvec{r}}} {\varvec{P}}_{{{\varvec{ij}}}}^{2} }}$$

#### Bacterial community diversity calculation

Based on the OTUs abundance matrix, the Shannon-Winner diversity indexes and OTUs species richness of each habitat-specialization subgroups and abundant subgroups were calculated. The non-metric multidimensional scaling (NMDS) function of ‘vegan’ package was used to analyze differences in bacterial subgroup community structure between afforestation types, and the significance of structural differences was tested by Permanova test (by ANOSIM function in vegan package of R software, permutations = 999, bray). Mantel test was carried out to analyze the relationship between community structure and environmental factors such as tree species composition, plant diversity, physicochemical properties and spatial distance. The spatial distance was calculated by the three-dimensional relationship of latitude coordinates, longitude coordinates and altitude. RDA function was taken to analyze the effects of tree composition, tree diversity and soil physicochemical properties on microbial subgroup community structure. The significance of RDA model was tested by ANOVA and the Monte Carlo permutation test was taken to analyze the significance of the influencing factors of each explanatory variable in the RDA model (by anova.cca and envfit function in vegan package of R software, permutations = 999). Plotting was done using the R 4.0.0 basic program package and ‘ggplot2’ package. The significance of soil physicochemical properties, niche width, abundance and α-diversity indexes among forest types were analyzed by one-way ANOVA test (SPSS 19.0).

## Results and analysis

A total of 2,506,761 sequences were tested from all soil samples, and were divided into 1830 OTUs. The sequences number of 20 topsoil samples ranged from 46,595 to 73,284, and the OTUs ranged from 940 to 1579. Sequences number of 20 subsoil samples ranged from 47,705 to 70,743, and OTUs ranged from 1181 to 1568. On the habitat-specialization gradient, the OTUs numbers of habitat-generalized subgroup accounted for only 5.19%, and the abundance ratio was only 1.70%. The habitat-specialized subgroup accounted for 65.41%, and the abundance ratio was also as high as 83.23% in topsoil. The OTUs number of the habitat-generalized subgroup accounted for only 5.79% and the abundance ratio was only 1.83%, while the OTUs number of habitat-specialized subgroup was as high as 62.79% and the abundance ratio was as high as 81.88% in subsoil. On the abundance gradient, the OTUs numbers of abundant subgroup in the topsoil accounted for only 3.20%, but the abundance ratio was as high as 47.38%. OTUs numbers of rare subgroup accounted for 93.82%, but the abundance ratio was only 41.21%. The OTUs numbers of abundant subgroup in subsoil only accounted for 1.87%, but the abundance ratio was as high as 36.55%. The OTUs numbers of rare subgroup accounted for as high as 94.84%, but the abundance ratio was only 47.22%. Soil physicochemical features were shown in Table 2.

**Table 2:** Soil physicochemical properties of each afforestation types.

	Forest type	Dry matter (g·kg^-1^)	Available Phosphorus (g·kg^-1^)	Organic Carbon (mg· kg^-1^)	NH_4_^+^-N (mg·kg^-1^)	NO_3_^-^-N (mg·kg^-1^)	NO_2_^-^-N (mg·kg^-1^)	pH
Topsoil	S	0.987 ± 0.002a	8.341 ± 1.069a	2.641 ± 0.688c	11.637 ± 1.212b	7.614 ± 0.934b	4.628 ± 3.044a	5.01 ± 0.157ab
	CF	0.982 ± 0.005a	3.273 ± 1.627b	4.032 ± 0.757a	11.551 ± 0.703b	8.443 ± 2.375a	4.221 ± 0.919a	4.693 ± 0.257b
	MF	0.983 ± 0.003a	3.923 ± 2.370b	3.943 ± 0.576b	12.692 ± 0.514b	7.240 ± 1.709b	4.561 ± 1.276a	4.727 ± 0.340b
	BF	0.978 ± 0.009b	2.614 ± 1.535c	3.496 ± 1.065b	14.121 ± 4.760a	7.304 ± 1.848b	3.970 ± 1.008b	5.274 ± 0.492a
Subsoil	S	0.988 ± 0.001a	2.523 ± 1.887b	1.539 ± 0.779b	8.986 ± 1.076b	5.655 ± 1.747b	2.874 ± 0.464b	5.017 ± 0.068a
	CF	0.986 ± 0.003a	3.005 ± 2.564b	2.568 ± 0.680a	11.970 ± 3.779a	7.101 ± 1.729a	3.683 ± 0.252a	4.767 ± 0.328a
	MF	0.986 ± 0.003a	1.903 ± 0.662c	1.778 ± 0.505b	10.633 ± 1.320a	5.767 ± 0.363b	3.292 ± 0.800a	4.837 ± 0.188a
	BF	0.984 ± 0.005a	3.294 ± 1.314a	2.060 ± 0.789ab	11.658 ± 3.992a	7.136 ± 2.916a	3.928 ± 2.020a	5.285 ± 0.416a

Different lowercase letters indicate that the index of the same soil layer is significantly different at the level of 0.05.

### Effects of afforestation types on the distribution frequency and abundance of soil bacteria along habitat-specialization and abundance gradients

**Figure 1:**
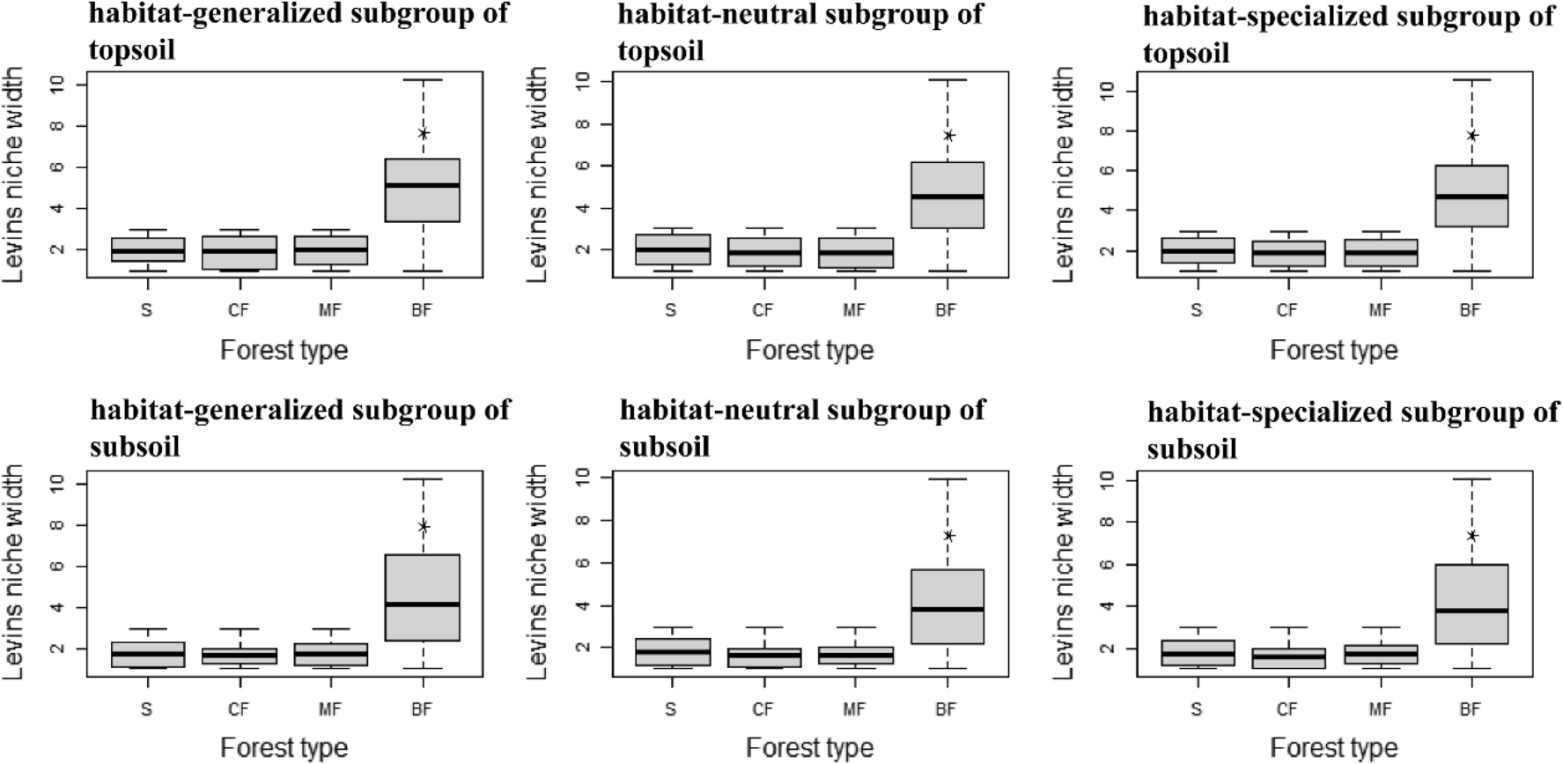
Effects of afforestation types on niche width of soil bacteria of different habitat-specialization subgroups (S: Shrub; CF: Coniferous forests; MF: Mixed coniferous and broad-leaved forest; BF: Broad-leaved forests; * indicates significant difference at the level of 0.01).

**Figure 2:**
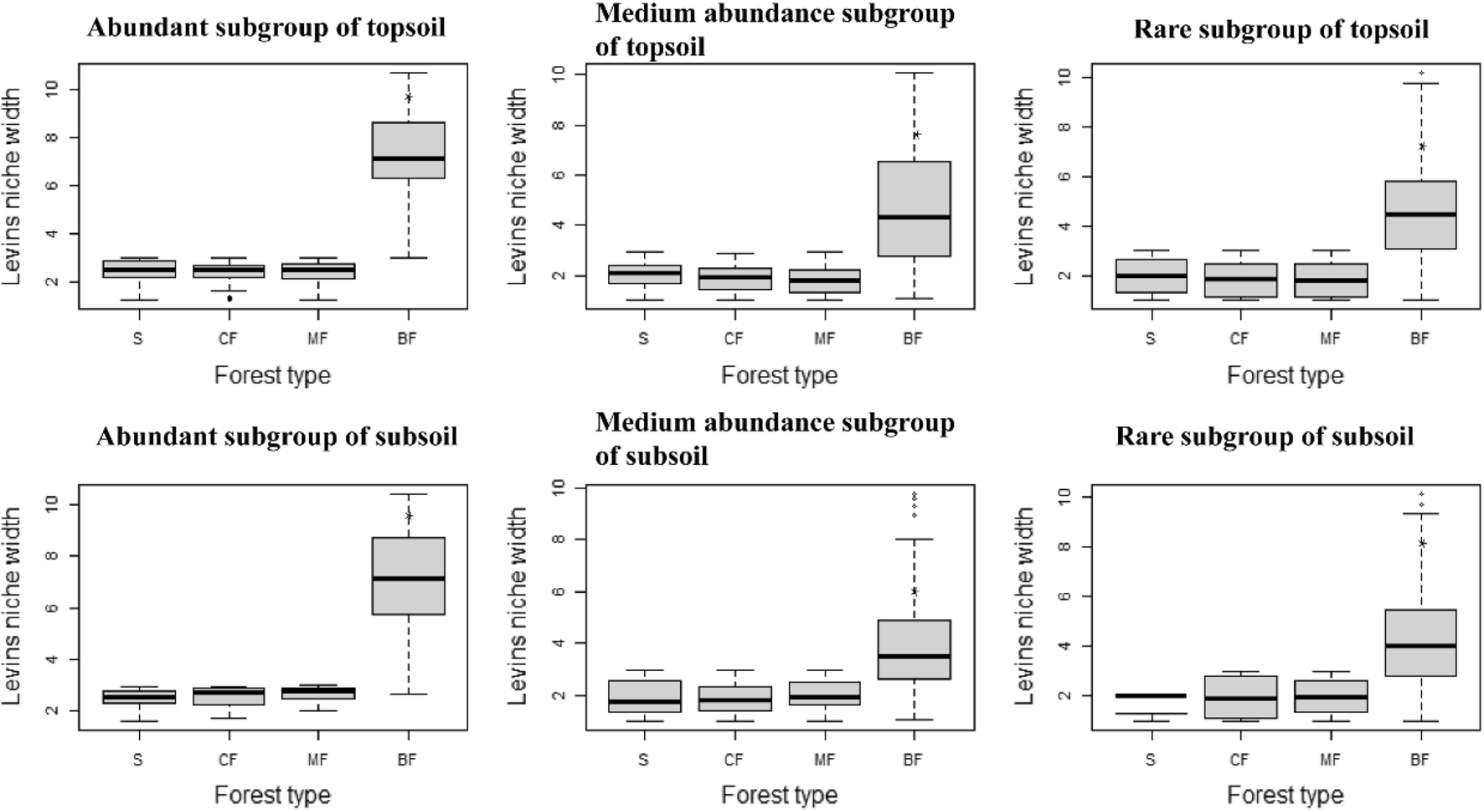
Effects of afforestation types on niche width of soil bacteria of different abundance subgroups (S: Shrub; CF: Coniferous forests; MF: Mixed coniferous and broad-leaved forest; BF: Broad-leaved forests; * indicates significant difference at the level of 0.01).

Distribution frequency of soil bacteria was presented by the niche width. Results showed the distribution frequency of each habitat-specialization subgroup in topsoil and subsoil was significantly the highest in broad-leaved forests (*P* < 0.01), and even the frequency of habitat-neutral and specialized subgroups in coniferous forests and mixed forests was slightly lower than in the shrub (Fig. 1). The distribution frequency of each abundance subgroup in topsoil and subsoil was also increased obviously in broad-leaved forests, especially for abundant subgroups (*P* < 0.01). The niche width of the abundant subgroup in broad-leaved forests also showed higher than other subgroups (Fig. 2).

**Figure 3:**
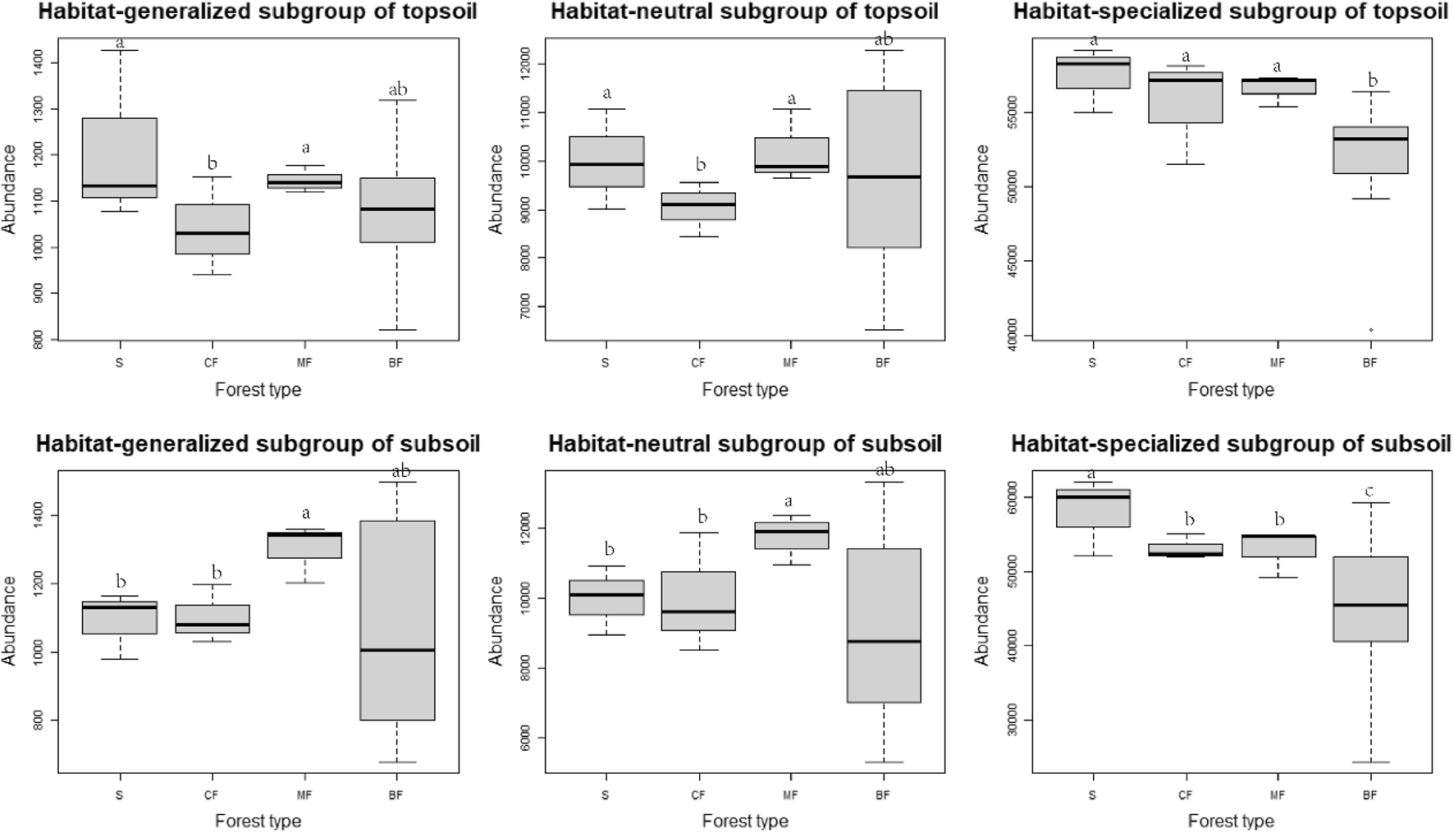
Effects of afforestation types on the abundance of soil bacteria of different habitat-specialization subgroups (S: Shrub; CF: Coniferous forests; MF: Mixed coniferous and broad-leaved forest; BF: Broad-leaved forests; Different lowercase letters indicate that the index of the same soil layer is significantly different at the level of 0.05).

**Figure 4:**
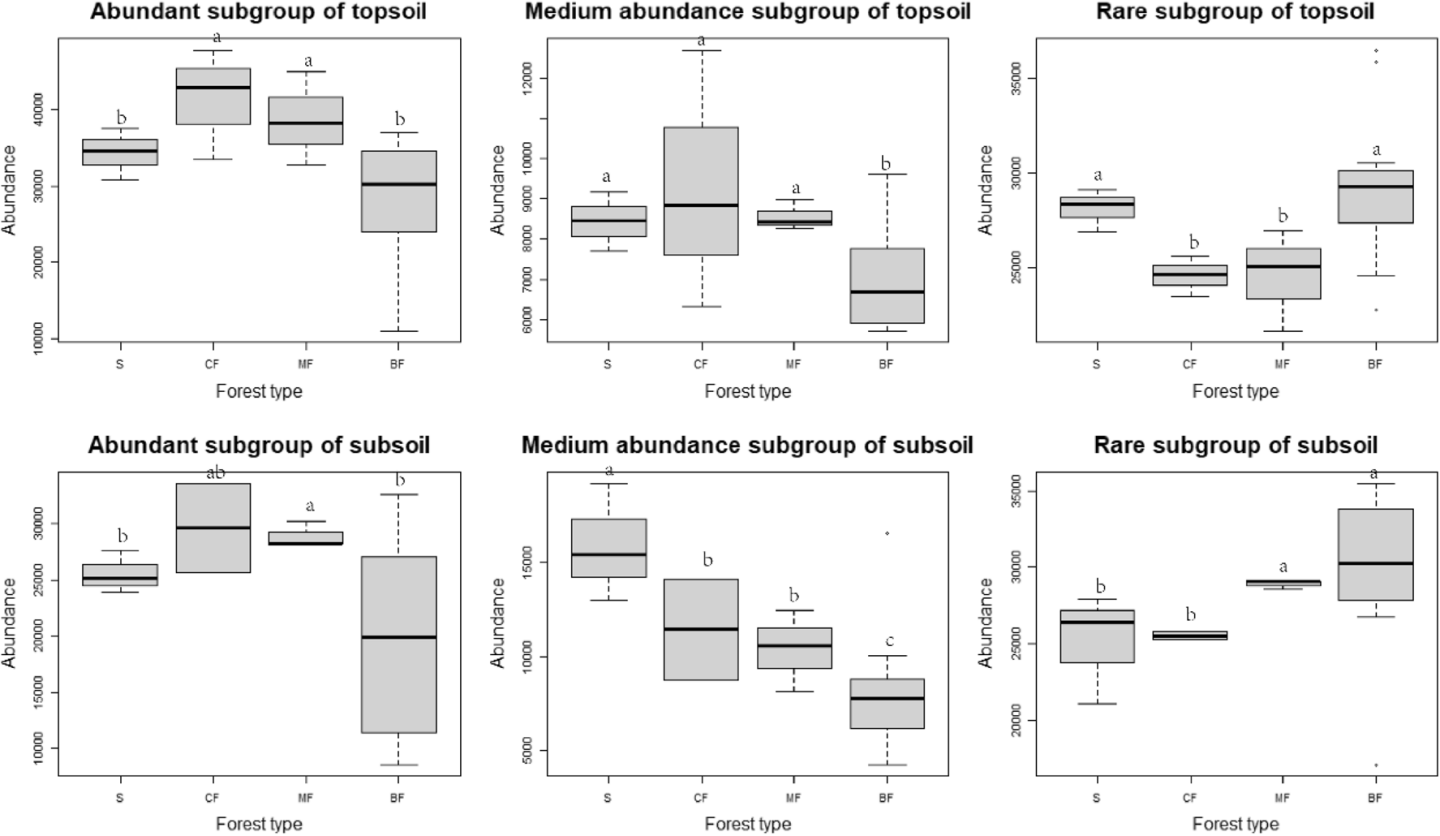
Effects of afforestation types on the abundance of soil bacteria of different abundance subgroups (S: Shrub; CF: Coniferous forests; MF: Mixed coniferous and broad-leaved forest; BF: Broad-leaved forests; Different lowercase letters indicate that the index of the same soil layer is significantly different at the level of 0.05).

On the habitat-specialization gradient, the abundance of each subgroup in topsoil has increased with habitat-specialization. The abundance of the same habitat-specialization subgroup in the topsoil and subsoil showed consistent with the stand changes. The abundance of habitat-generalized and neutral subgroups was higher in shrub and mixed forests than in the coniferous forests and broad-leaved forests and that of habitat-specialized subgroup showed the highest in the shrub and the lowest in the broad-leaved forests, and significantly differ (*P* < 0.05) (Fig. 3). On the abundance gradient, the abundance of abundant and medium abundance subgroups in the topsoil and subsoil both significantly decreased, whereas that of rare subgroup increased in broad-leaved forests. This indicated the soil conditions of broad-leaved forests were more comfortable for most species of the rare subgroup. The abundance of abundant subgroup of both soil layer in coniferous forests and mixed forests were significantly higher than in shrub and broad-leaved forests. The abundance of medium abundance subgroup was lower in the topsoil of broad-leaved forests than in other stands, but obviously the trend of shrub > coniferous forests > mixed forests > broad-leaved forests in the subsoil. The abundance features of rare subgroup in topsoil were opposite to those of abundant subgroup (Fig. 4).

### Effects of afforestation types on soil bacterial α-diversity characteristics along habitat-specialization and abundance gradients

**Figure 5:**
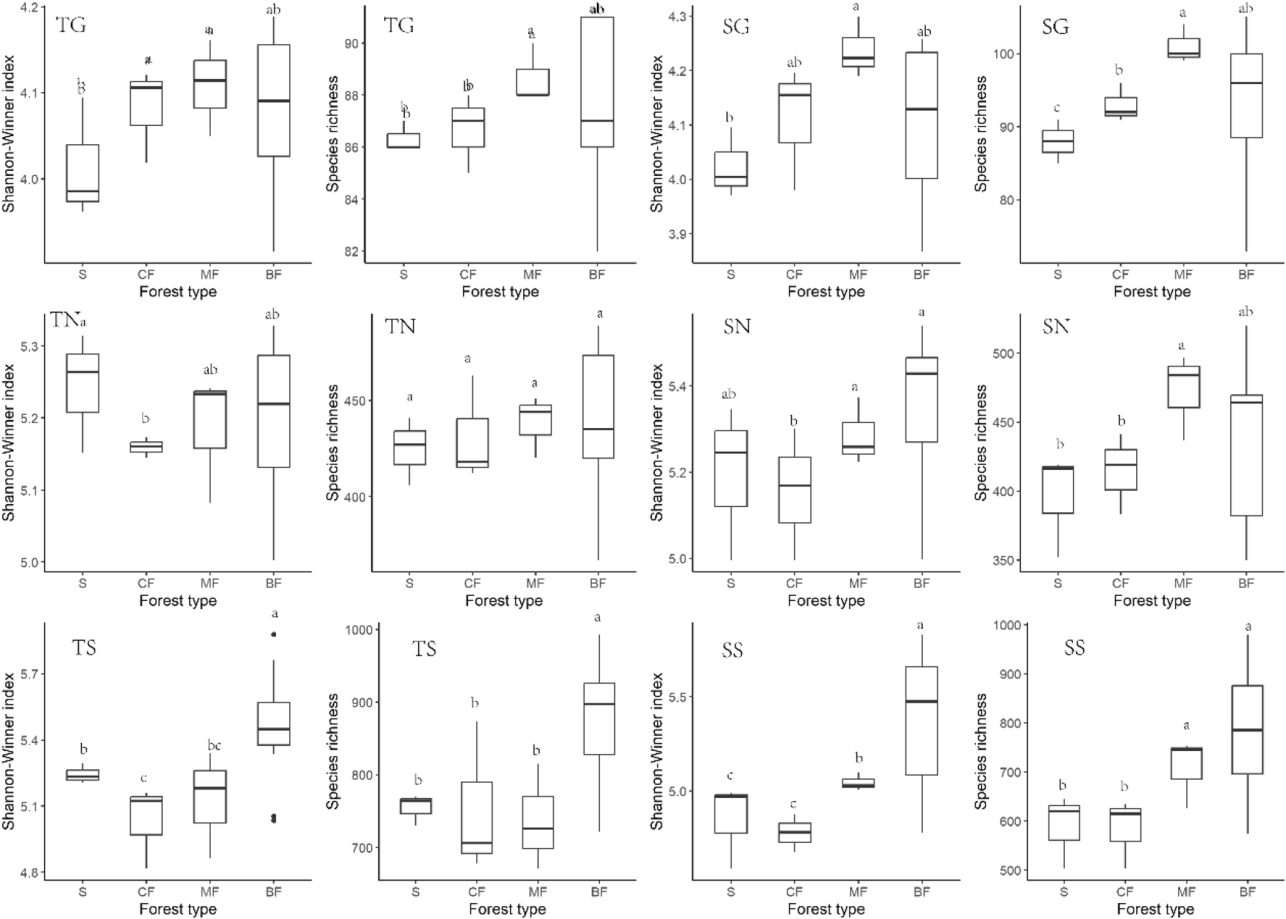
Effects of afforestation types on α diversity of soil bacteria of different habitat-specialization subgroups (S: Shrub; CF: Coniferous forests; MF: Mixed coniferous and broad-leaved forest; BF: Broad-leaved forests; TG: Habitat-generalized subgroup of topsoil; TN: Habitat-neutral subgroup of topsoil; TS: Habitat-specialized subgroup of topsoil; SG: Habitat-generalized subgroup of subsoil; SN: Habitat-neutral subgroup of subsoil; SS: Habitat-specialized subgroup of subsoil; Different lowercase letters indicate that the index of the same soil layer is significantly different at the level of 0.05).

**Figure 6:**
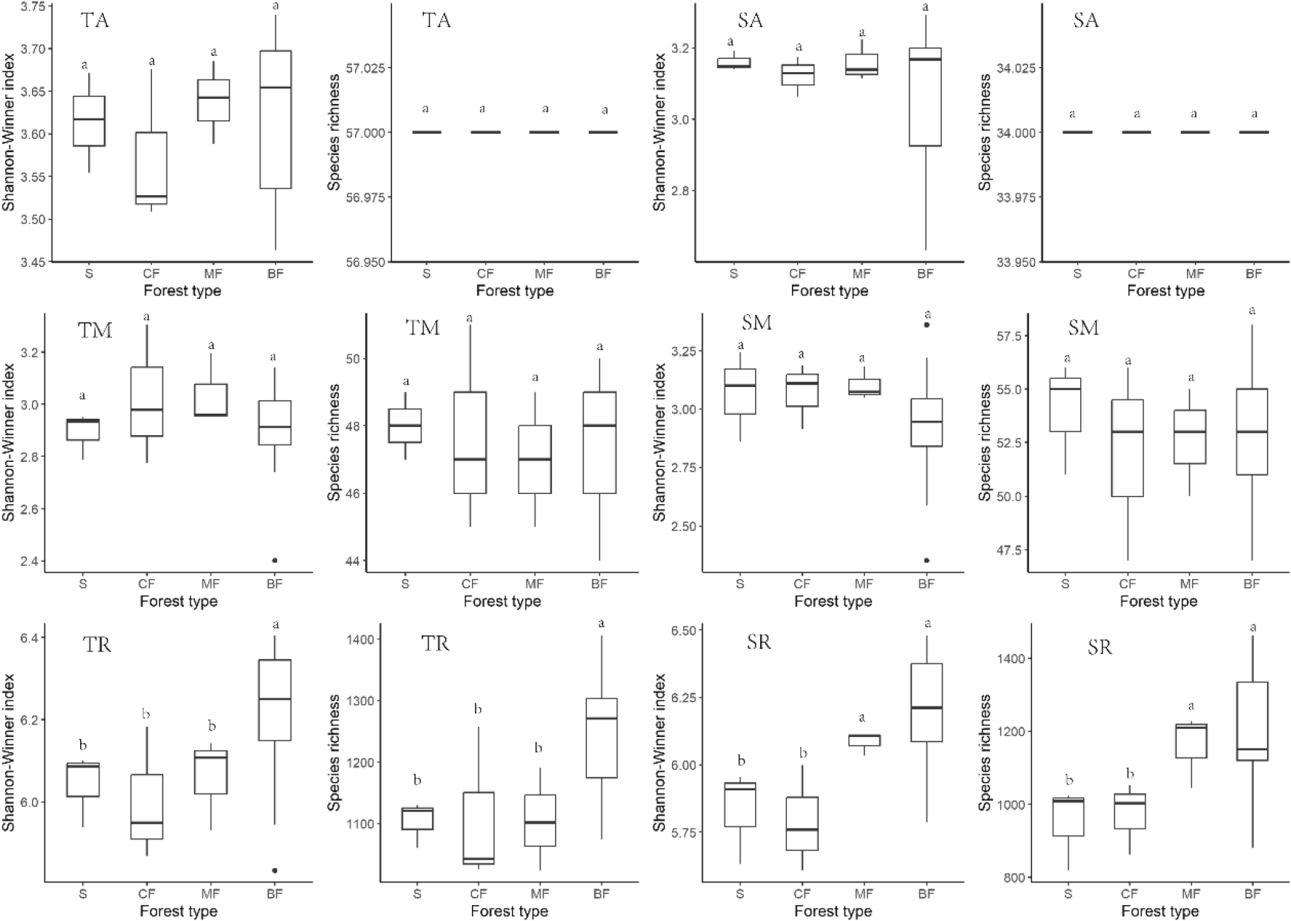
Effects of afforestation types on α diversity of soil bacteria of different abundance subgroups (S: Shrub; CF: Coniferous forests; MF: Mixed coniferous and broad-leaved forest; BF: Broad-leaved forests TA: Abundant subgroup of topsoil; TM: Medium abundance subgroup of topsoil; TR: Rare subgroup of topsoil; SA: Abundance subgroup of subsoil; SM: Medium abundance subgroup of subsoil; SR: Rare subgroup of subsoil; Different lowercase letters indicate that the index of the same soil layer is significantly different at the level of 0.05).

Shannon-Winner index of habitat-generalized subgroup of each afforestation stands in both topsoil and subsoil was significantly higher than that of shrub in all tree forests (*P* < 0.05) (Fig. 5(TG)(SG)), but only the index of habitat-specialized and neutral subgroups in subsoil of broad-leaved forests significantly higher than other stands (*P* < 0.05) (Fig. 5(TS)(SS)), and the habitat-neutral subgroup of topsoil were almost unaffected by afforestation (Fig. 5(TN)). The species richness of habitat-specialized subgroup in topsoil was significantly higher in broad-leaved forests than in other stands (*P* < 0.01) (Fig. 5(TN)), while that of habitat-generalized and neutral subgroups was highest in mixed forests (Fig. 5(TG)(TN)). The species richness of each habitat-specialization subgroups in subsoil was higher in broad-leaved forests and mixed forests than in coniferous forests and shrub (Fig. 5(SG)(SN)(SS)). The diversity of rare subgroup in both soil layer was higher than that of abundant and medium abundance subgroups. Shannon-Winner index of abundant and rare subgroups in topsoil and subsoil was higher in broad-leaved forests and mixed forests than in coniferous forests and shrub (Fig. 6(TA)(SA)(TR)(SR)). Species richness of topsoil rare subgroup was significantly higher in broad-leaved forests than in other stands, and that of rare subgroup in subsoil was significantly higher in broad-leaved forests and mixed forests than in coniferous forests and shrub (*P* < 0.05). Shannon-diversity index and species richness were consistent among forest types in both topsoil and subsoil (Fig. 6(TM)(SM)).

### Responses and driving factors of soil bacterial composition and structure to afforestation types along habitat-specialization and abundance gradients

**Figure 7:**
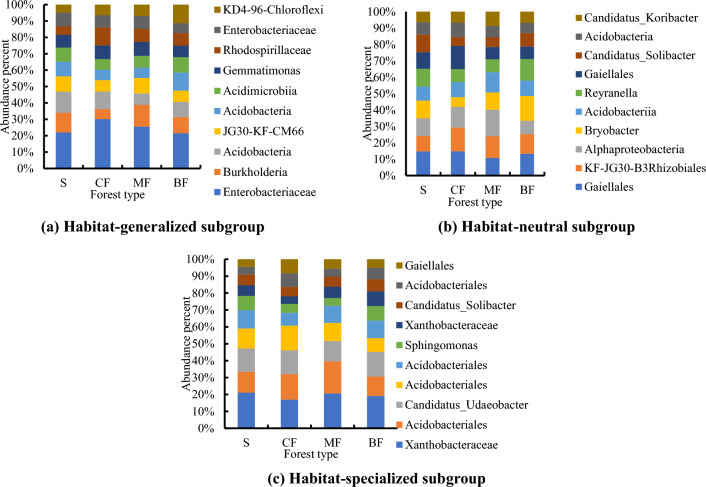
Composition of bacterial OTUs of different habitat-specialization subgroups of topsoil (S: Shrub; CF: Coniferous forests; MF: Mixed coniferous and broad-leaved forest; BF: Broad-leaved forests).

**Figure 8:**
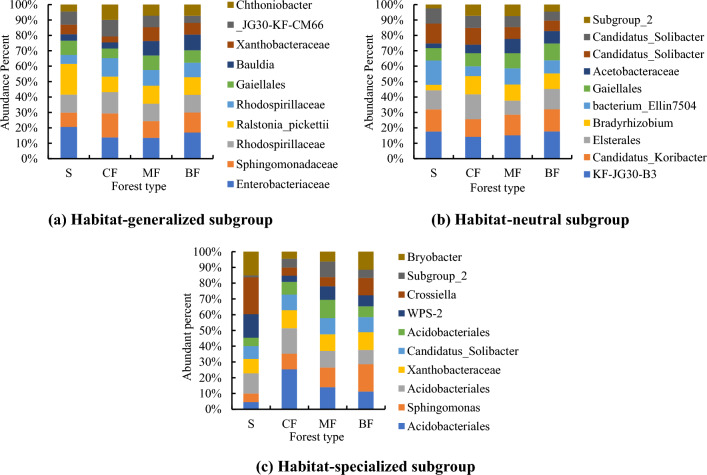
Composition of bacterial OTUs of different habitat-specialization subgroups of subsoil (S: Shrub; CF: Coniferous forests; MF: Mixed coniferous and broad-leaved forest; BF: Broad-leaved forests).

For OTUs with abundance greater than 1%, species number of habitat-generalized subgroup was significantly higher than that of habitat-neutral and specialized subgroups, and the abundance distribution was more uniform in the topsoil (Fig. 7a, b, c). Among all forest types, the distribution uniformity of abundance ratio was the best in broad-leaved forests. The abundance ratio of top 3 OTUs in topsoil habitat-generalized and neutral subgroups were the highest in the coniferous forests (Fig. 7a, b), while the highest of specialized subgroup was in the shrub (Fig. 7a). The abundance ratio of each OTUs of all habitat-specialization subgroups in broad-leaved forests was the lowest, which was consistent with its higher distribution uniformity. The abundance ratio of top 3 OTUs of habitat-generalized subgroup was the highest in shrub, and the highest of the habitat-neutral and specialized subgroups was in the coniferous forests (Fig. 8a, b, c).

**Figure 9:**
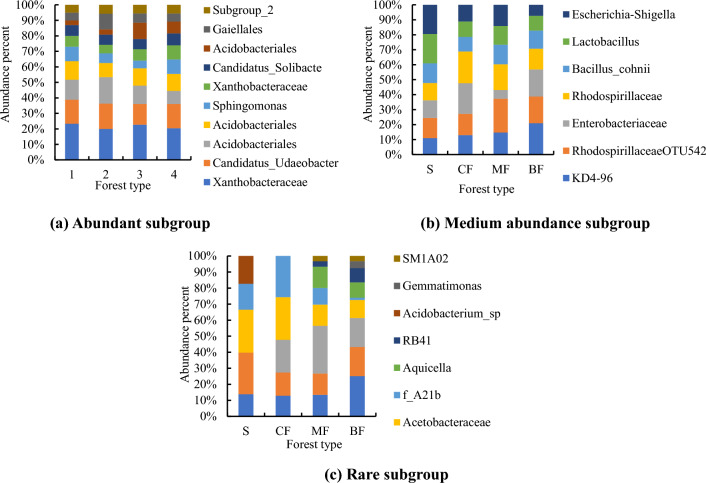
Composition of bacterial OTUs of different abundance subgroups of topsoil (S: Shrub; CF: Coniferous forests; MF Mixed coniferous and broad-leaved forest; BF: Broad-leaved forests).

**Figure 10:**
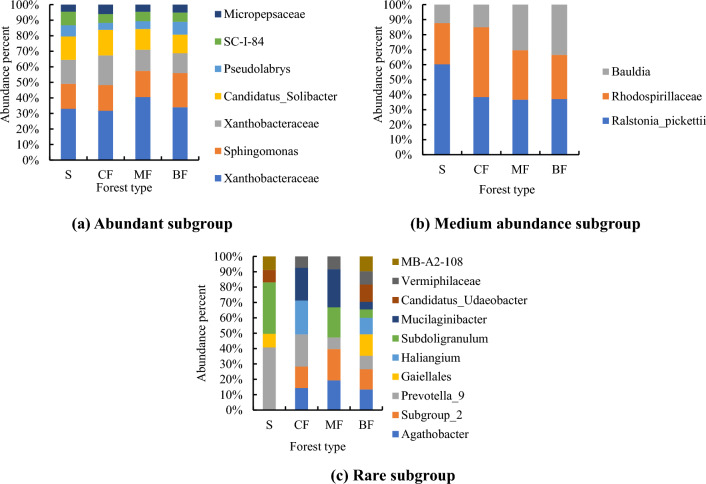
Composition of bacterial OTUs of different abundance subgroups of subsoil (S: Shrub; CF: Coniferous forests; MF: Mixed coniferous and broad-leaved forest; BF: Broad-leaved forests).

Among the top three abundant subgroups of topsoil, the highest abundance ratio was found in the coniferous forests (Fig. 9a), the highest abundance of medium abundance subgroup was found in the mixed forests and the highest in shrub of rare subgroup (Fig. 9b), and the highest abundance was found in the shrub layer in the rare groups (Fig. 9c). In subsoil, the highest proportion of OTUs of abundant subgroup was found in mixed forests (Fig. 10a), and that of medium abundance and rare subgroups was found in shrub (Fig. 10b, c).

**Figure 11:**
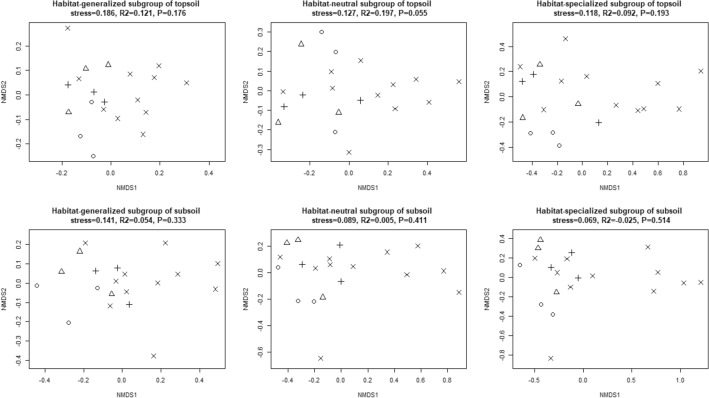
NMDS 2D figure of effects of afforestation types on bacterial community structure of each habitat-specialization subgroup (○ represents shrub; △ represents coniferous forests; + represents mixed coniferous and broad-leaved forests; × represents broad-leaved forests).

**Figure 12:**
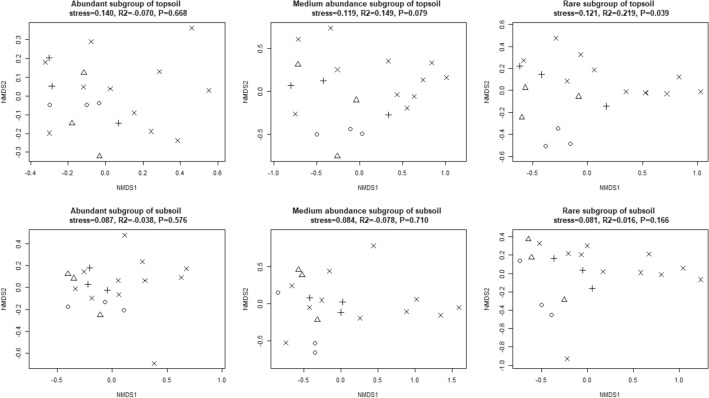
NMDS 2D figure of effects of afforestation types of bacterial community structure of each abundance subgroup (○ represents shrub; △ represents coniferous forests; + represents mixed coniferous and broad-leaved forests; × represents broad-leaved forests).

NMDS analysis was carried out on the community structural difference among afforestation types along habitat-specialization and abundance gradient. The 2D figures showed that the coordinate points of the rare subgroup in both layers of the broad-leaved forests were significantly separated from other stands, that is, the coordinate points of the broad-leaved forests were distributed at the upper right position of the figure, while that of other stands distributed at the lower left. The coordinate points of community structure of each habitat-specialization subgroup could not be distinguished among each stand, and the same for abundant and medium abundance subgroups (Figs. 11, 12). However, in the right half of all NMDS maps, only coordinate points of broad-leaved forests were distributed, indicating that in some broad-leaved forests, bacterial community structure had been significantly different from that of other stands (Fig. 11). Further ANOSIM analysis showed that afforestation affected non-significantly on the community structure of each habitat-specialization subgroup in both topsoil and subsoil, but significantly affected the community structure of the rare subgroup in topsoil.

The environmental affection on the community structure among afforestation types of each subgroup have also been analyzed. Mantel analysis showed that plant diversity and vegetation species composition rather than soil chemical properties and spatial distance significantly affected bacterial community structure among habitat-specialization and abundant subgroups. Plant diversity significantly affected the structure of each habitat-specialization subgroup among afforestation types in both layers, and the species composition of tree layer significantly affected the structure of each habitat-specialization subgroup in topsoil and habitat-specialized subgroup in subsoil. The species composition of shrub layer significantly affected the structure of habitat-specialized subgroup in topsoil (Table 3). The species composition of tree layer significantly affected the structure of all abundance subgroups except abundant subgroup of subsoil. Plant diversity and species composition of shrub layer significantly affected the structure of medium abundance and rare subgroups in topsoil (Table 4).

**Table 3:** Mantel test of effect of environmental factors on soil bacterial community structure of each habitat-specialization subgroup.

Factor	TG		TN		TS		SG		SN		SS	
	R	P	R	P	R	P	R	P	R	P	R	P
Soil chemical properties	0.028	0.389	0.063	0.298	0.079	0.201	0.088	0.297	− 0.004	0.483	0.047	0.341
Plant diversity	0.213	**0.019**	0.301	**0.002**	0.272	**0.008**	0.228	**0.017**	0.202	**0.022**	0.203	**0.027**
Spatial distance	− 0.073	0.691	− 0.053	0.685	− 0.043	0.648	− 0.054	0.615	− 0.036	0.572	− 0.039	0.600
Tree species	0.213	**0.023**	0.189	**0.041**	0.276	**0.005**	0.101	0.183	0.206	0.053	0.241	**0.024**
Shrub species	0.127	0.190	0.158	0.102	0.226	**0.021**	0.121	0.167	0.175	0.103	0.201	0.075

*TG* habitat-generalized subgroup of topsoil, *TN* habitat-neutral subgroup of topsoil, *TS* habitat-specialized subgroup of topsoil, *SG* habitat-generalized subgroup of subsoil, *SN* habitat-neutral subgroup of subsoil, *SS* habitat-specialized subgroup of subsoil.

Bold indicates *P* < 0.05.

**Table 4:** Mantel test of effect of environmental factors on soil bacterial community structure of each abundance subgroup.

Factor	TA		TM		TR		SA		SM		SR	
	R	P	R	P	R	P	R	P	R	P	R	P
Soil chemical properties	0.040	0.379	0.146	0.074	0.039	0.360	0.034	0.362	0.068	0.271	0.003	0.460
Plant diversity	0.100	0.147	0.269	**0.006**	0.261	**0.002**	0.216	**0.022**	0.112	0.120	0.214	**0.018**
Spatial distance	0.014	0.504	− 0.125	0.914	− 0.017	0.546	− 0.061	0.611	− 0.003	0.455	− 0.001	0.466
Tree species	0.327	**0.003**	0.175	**0.032**	0.234	**0.007**	0.202	0.056	0.266	**0.014**	0.196	**0.031**
Shrub species	0.138	0.127	0.194	**0.018**	0.214	**0.011**	0.180	0.121	0.229	**0.038**	0.195	0.055

*TA* abundant subgroup of topsoil, *TM* medium abundance subgroup of topsoil, *TR* rare subgroup of topsoil, *SA* abundance subgroup of subsoil, *SM* medium abundance subgroup of subsoil, *SR* rare subgroup of subsoil.

Bold indicates *P* < 0.05.

### RDA analysis of effects of vegetation and environmental factors on soil bacterial structure along habitat-specialization and richness gradients

#### Effect of species composition

On the habitat-specialization gradient, the RDA model of whole species composition of tree layer significantly explained the community structure of each habitat-specialization subgroup in both topsoil and subsoil. But the change of important value of any individual tree species did not show significant effect in the topsoil. The importance values of *Robinia pseudoacacia* and *Morus alba* significantly affected the community structure of the habitat-specialized subgroup in subsoil (Table S1, Fig. S1). The RDA model of species composition of shrub layer significantly explained the community structure of habitat-generalized and neutral subgroups in the subsoil. Individual shrub species composition did not significantly affect the community structure of habitat-generalized subgroup in the topsoil. The significant impact factors for habitat-neutral subgroup in topsoil was *Platycladus orientalis*, *Robinia pseudoacacia* and *Morus mongolica*, for habitat-specialized subgroup in topsoil was *Vitex negundo* var. *heterophylla*, for habitat-generalized subgroup in subsoil was *Vitex negundo* var. *heterophylla* and *Vitex negundo* var. *heterophylla* and for habitat-neutral and specialized subgroups in subsoil was *Robinia pseudoacacia* and *Morus mongolica* (Table S2, Fig. S2).

On the abundance gradient, the RDA models of overall species composition of tree layer for explaining the community structure of medium abundance and rare subgroups of topsoil as well as abundant and rare subgroups of subsoil were significant. In the topsoil, the significant impact species for abundant subgroup were of *Quercus acutissima* and *Pinus densiflora*, for medium abundance subgroup was *Populus davidiana*, *Diospyros lotus* and for rare subgroup was *Robinia pseudoacacia* and *Populus davidiana*. In the subsoil, significant impact factors of abundant subgroup were *Platycladus orientalis*, *Robinia pseudoacacia*, *Morus alba* and *Ziziphus mauritiana*, and for medium abundance subgroup were *Robinia pseudoacacia* and *Morus alba* (Table S3, Fig. S3). The overall species composition of shrub layer could not significantly explain the changes of community structure of each abundance subgroup. Monte Carlo analysis also showed that only *Vitex negundo* var. *heterophylla*, *Vitex negundo* var. *heterophylla* and *Morus mongolica* significantly affected the community structure of a few subgroups (Table S4, Fig. S4).

#### Effect of plant diversity

The RDA model of plant diversity feature to explain the community structure of each habitat-specialization subgroup in the topsoil and subsoil was significant. Monte Carlo test results has also showed that all the diversity indices significantly affected the structure of each subgroup in the topsoil, and the evenness indices significantly affected the structure of each subgroup in the subsoil (Table S5, Fig. S5). For each abundance subgroup, all diversity indexes have significantly affected the structure changes of rare subgroup in topsoil and medium abundance subgroups in subsoil. Shannon-Winner diversity index, Simpson diversity index and Pielou evenness index had significant effects on the structure of other subgroups in topsoil and subsoil (Table S6, Fig. S6).

#### Effect of soil chemical properties

The RDA model of soil physicochemical properties to explain the community structure of habitat-neutral and specialized subgroups in topsoil and habitat-generalized subgroup in subsoil was significant. However, Monte Carlo test showed that the significant factors affecting the structure of each subgroup were different (Table S7). According to the RDA 2D figure, the differences of each habitat-specialization subgroup in the topsoil has been more affected by soil physicochemical properties than that in the subsoil. The coordinates community structure of the habitat-generalized and neutral subgroups of broad-leaved forests in topsoil directed the same as the increase of pH, available P and ammonia nitrogen, indicating that these physicochemical properties have significantly changed the structure of the two subgroups of in the topsoil of broad-leaved forests, while the coordinate points of the habitat-specialized subgroup in subsoil of broad-leaved forests were contrary to the changes of ammonia nitrogen and pH, indicating that ammonia nitrogen and pH also significantly affected the structure habitat-specialized subgroup in subsoil. No significant difference has been proved in the structure of other subgroups among stands (Fig. S7). For each abundance subgroup, soil physicochemical properties significantly explained the changes of community structure of rare subgroup in topsoil. Monte Carlo test showed the structure of abundant subgroup significantly correlated with the content of available P and nitrate nitrogen, and that of medium abundance and rare subgroup significantly correlated with the content of ammonia nitrogen and pH. In the subsoil, the structure of abundant subgroup was significantly correlated with ammonia nitrogen content, and that of medium abundance subgroup was significantly correlated with dry matter content and ammonia nitrogen content, as well as the structure of rare subgroup was significantly correlated with dry matter, organic carbon, nitrate nitrogen content and pH (Table S8, Fig. S8).

## Discussion

### Response of abundance and distribution frequency of soil bacteria to afforestation along habitat-specialization and abundance gradients

Research results have shown that the consistency of variation of species number and abundance of soil bacterial OTUs were significantly different on the two gradients. At the habitat-specialization gradient, the species number and abundance ratio changed consistently and showed the highest of habitat-specialized subgroup while the lowest of habitat-generalized subgroup. This was consistent with the research of Estavillo et al. about forest loss and biodiversity threshold^57^. However, at the abundance gradient, the abundant subgroup owns a small number of OTUs species and a high abundance ratio, while the rare subgroup showed the opposite. Some studies also showed that the total number of sequences of abundant subgroup was dominant while the species number of rare subgroup was absolutely dominant^18^.

The distribution frequency of soil bacterial OTUs was characterized by Levins niche width. The higher the value is, the more evenly distributed the individuals are in each resource state, that is, the higher the degree of niche differentiation and the better the interspecies co-existence. However, a lower value means that the distribution of individuals in each resource state is different and the co-existence between species is bad. Our results showed that the response of distribution frequency to stand change was highly consistent between the two subgroups classification gradients. The broad-leaved forests significantly promoted the coexistence and niche differentiation of each habitat-specialization subgroup, and most positively promoted the distribution frequency of abundant subgroup on the abundance gradient. The niche width of each habitat-specialization subgroup in the same forest type were similar, but that of the abundant subgroup were higher than the medium abundance and rare subgroups, especially in the broad-leaved forests. This was because abundant subgroup existed more abundant-dominant OTUs than rare subgroup, allowing them more competitive and thus adapt quickly to changing environments^54^.

The response of bacterial OTUs abundance to stand changes also differ between the two gradients. The abundance of each habitat-specialization subgroup was the lowest in the broad-leaved forests, which could be interpreted as that the soil environment of broad-leaved forests was conducive to niche differentiation among species and reduced the occurrence probability of a few high-abundance species^58^. The abundance of abundant and medium abundance subgroups in broad-leaved forests also decreased, but the abundance of rare subgroup increased, which could be interpreted as the decline in abundance of abundant subgroup enhanced the abundance of rare subgroup and occupied new niches^59,60^.

### Response of α diversity characteristics to afforestation along habitat-specialization and abundance gradients

The α diversity of habitat-specialized subgroup and rare subgroup was the highest on their respective gradients. This was consistent with the conclusion reported by Wu et al. that the diversity and species richness of rare subgroup in topsoil and subsoil were significantly higher than those of abundant and medium abundance subgroups^58^. In general, the diversity index of each subgroup in broadleaved forests was higher than that of other stands^61^, and the response of diversity to stand change on habitat-specialization gradient was stronger than that on abundance gradient, of which the habitat-specialized subgroup responded the strongest. A number of studies have shown that bacterial habitat specialists were more susceptible to extinction than bacterial habitat generalists when habitat conditions changed^62,63^, and this could legitimately interpret the fact that bacterial α-diversity of habitat-specialization gradient more strongly responded to stand changes than the abundance gradient. The abundance gradient only represents the proportion of individual number of species, and the response of species and individual numbers of each subgroup to stand change were similar and intersecting, so the response of α diversity to stand change on the abundance gradient was weak.

### Responses of soil bacterial composition and community structure to afforestation along habitat-specialization and abundance gradients

The results showed that the response of the composition and distribution features of bacteria to stand change was different between two classification gradients. In the broadleaved forests, the distribution uniformity of each habitat-specialization subgroup was the highest. However, on the abundance gradient, the most uniform distribution of OTUs in the broad-leaved forests was only in the abundant and rare subgroup of topsoil and rare subgroup of subsoil. This was consistent with the fact that rare subgroup owns a large number of species but a low abundance, which resulted in a more even distribution of abundance than the abundant and medium abundance subgroups^64,65^. In addition, the most uniform distribution in broad-leaved forests and the worst distribution uniformity in shrub and coniferous forests, was consistent with the conclusion on the increasing complexity of soil bacterial network from temperate coniferous forests succession to broad-leaved forests in Liupanshan Mountain^66^, which promoted co-existence and niche differentiation by reducing intraspecific and interspecific competition of bacteria^67^. Coordinate points of community structure of each habitat-specialization subgroup of some broad-leaved forests were obviously seperated from that of other stands. These indicated the soil environmental changes in broad-leaved forests lead to significant changes in soil bacterial community structure on the gradient of habitat-specialization. This conclusion was consistent with that the distribution frequency of each habitat-specialization subgroup in broad-leaved forests significantly higher than other stands thus the community structure must be obviously different from that of other stands in which a few OTUs dominated. In terms of abundance gradient, ANOSIM analysis showed that the differences among each forest type only significantly affected the community structure of rare subgroup in the topsoil, because their niche width was narrow and more sensitive to environmental changes^68^, while the abundant subgroup tended to be more resilient and robust to environmental changes. The conclusion of this study provides a scientific reference and a new perspective for the comparative study of soil microbial response to stand change from the two gradients of soil microbial habitat specialization and abundance.

### Supplementary Information

Supplementary Information.

## Data Availability

SRA: https://www.ncbi.nlm.nih.gov/bioproject/PRJNA852687.

## References

[1] Sasmito SD, Kuzyakov Y, Lubis AA, Murdiyarso D, Hutley LB, Bachri S, Friess DA, Martius C, Borchard N (2020). Organic carbon burial and sources in soils of coastal mudflat and mangrove ecosystems. CATENA.

[2] Tian D, Xiang W, Chen X (2011). A long-term evaluation of biomass production in first and second rotations of Chinese fir plantations at the same site. Forestry.

[3] Jacobson S (2003). Addition of stabilized wood ashes to Swedish coniferous stands on mineral soils-Effects on stem growth and needle nutrient concentrations. Silva Fenn..

[4] Li JH (1987). Historical evolution of forests in Shandong Province Agricultural. Archaeology.

[5] Tan LC, Liu W, Wang TL, Cheng P, Zang JJ, Wang XQ, Ma L, Li D, Lan JH, Ai SB, Cheng H, Xu H, Ai L, Gao YL, Cai YJ (2020). A multiple-proxy stalagmite record reveals historical deforestation in central Shandong, northern China. Sci. Sin..

[6] Du ZY, Liang Y, Ge ZQ, Li ZT, Li YT, Lyn LC, Wang QH (2020). Soil quality characteristics of *Platycladus orientalis* plantations with different densities in central mountainous area of Shandong Province. J. Cent. South Univ. For. Technol..

[7] Jiao S, Chen WM, Wang JL, Du NN, Li QP, Wei GH (2018). Soil microbiomes with distinct assemblies through vertical soil profiles drive the cycling of multiple nutrients in reforested ecosystems. Microbiome.

[8] Wagg C, Schlaeppi K, Banerjee S, Kuramae EE, van der Heijden MGA (2019). Fungal-bacterial diversity and microbiome complexity predict ecosystem functioning. Nat. Commun..

[9] Baldrian P (2017). Forest microbiome: Diversity, complexity and dynamics. FEMS Microbiol. Rev.

[10] Yao MJ, Rui JP, Niu HS, Hedˇenec P, Li JB, He ZL, Wang JM, Cao WD, Li XZ (2017). The differentiation of soil prokaryote communities along a precipitation and temperature gradient in the eastern Inner Mongolia steppe. Catena.

[11] Geml J, Morgado LN, Semenova-Nelsen TA (2017). Changes in richness and community composition of ectomycorrhizal fungi among altitudinal vegetation types on Mount Kinabalu in Borneo. New Phytol..

[12] Wu B, Tian J, Bai C, Xiang M, Sun J, Liu X (2013). The biogeography of fungal communities in wetland sediments along the Changjiang River and other sites in China. ISME J..

[13] Canini F, Zucconi L, Pacelli C, Selbmann L, Onofri S, Geml J (2019). Vegetation, pH and water content as main factors for shaping fungal richness, community composition and functional guilds distribution in soils of western Greenland. Front. Microbiol..

[14] Porazinska DL, Farrer EC, Spasojevic MJ (2018). Plant diversity and density predict belowground diversity and function in an early successional alpine ecosystem. Ecology.

[15] Horrocks CA, Arango J, Arevalo A (2019). Smart forage selection could significantly improve soil health in the tropics. Sci. Total Environ..

[16] Qu Z, Liu B, Ma Y (2020). The response of the soil bacterial community and function to forest succession caused by forest disease. Funct. Ecol..

[17] Yang K, Zhu J (2015). The effects of N and P additions on soil microbial properties in paired stands of temperate secondary forests and adjacent larch plantations in Northeast China. Soil Biol. Biochem..

[18] Wu W, Wang X, Ren Z, Zhou X, Du G (2022). N-induced species loss dampened by clipping mainly through suppressing dominant species in an alpine meadow. Front. Plant Sci..

[19] Zhalnina K, Dias R, de Quadros PD (2015). Soil pH determines microbial diversity and composition in the park grass experiment. Microb. Ecol..

[20] Wan XH, Huang ZQ, He ZM (2015). Soil C: N ratio is the major determinant of soil microbial community structure in subtropical coniferous and broadleaf forest plantations. Plant Soil.

[21] Vuong TMD, Zeng JY, Man XL (2020). Soil fungal and bacterial communities in southern boreal forests of the Greater Khingan Mountains and their relationship with soil properties. Sci. Rep..

[22] Lange M, Habekost M, Eisenhauer N, Roscher C, Bessier H, Engels C, Oelmann Y, Scheu S, Wilcke W, Schulze ED, Gleixner G (2014). Biotic and abiotic properties mediating plant diversity effects on soil microbial communities in an experimental grassland. PLoS One.

[23] Lange M, Eisenhauer N, Sierra CA, Bessler H, Engels C, Griffiths RI, MelladoVazquez PG, Malik AA, Roy J, Scheu S, Steinbeiss S, Thomson BC, Trumbore SE, Gleixner G (2015). Plant diversity increases soil microbial activity and soil carbon storage. Nat. Commun..

[24] Wang H, Liu SR, Wang JX (2018). Mixed-species plantation with *Pinus massoniana* and *Castanopsis hystrix* accelerates C loss in recalcitrant coniferous litter but slows C loss in labile broadleaf litter in southern China. For. Ecol. Manag..

[25] Wu YC, Zeng J, Zhu QH (2017). pH is the primary determinant of the bacterial community structure in agricultural soils impacted by polycyclic aromatic hydrocarbon pollution. Sci. Rep..

[26] Liu LM, Yang J, Yu Z, Wilkinson DM (2015). The biogeography of abundant and rare bacterioplankton in the lakes and reservoirs of China. ISME J..

[27] Gravel D, Canham CD, Beaudet M (2006). Reconciling niche and neutrality: The continuum hypothesis. Ecol. Lett..

[28] Mueller RC, Paula FS, Mirza BS, Rodrigues JLM, Nusslein K, Bohannan BJM (2014). Links between plant and fungal communities across a deforestation chronosequence in the Amazon rainforest. ISME J..

[29] Han W, Wang G, Liu J (2021). Effects of vegetation type, season, and soil properties on soil microbial community in subtropical forests. Appl. Soil Ecol..

[30] Nielsen UN, Osler GHR, Campbell CD, Burslem DFRP, Wal RVD (2010). The influence of vegetation type, soil properties and precipitation on the composition of soil mite and microbial communities at the landscape scale. J. Biogeogr..

[31] Xu HD, Yu MK, Cheng XR (2021). Abundant fungal and rare bacterial taxa jointly reveal soil nutrient cycling and multifunctionality in uneven-aged mixed plantations. Ecol. Indic..

[32] Yin Y, Li Q, Du H (2021). Near-natural transformation of *Pinus tabuliformis* better improve soil nutrients and soil microbial community. Peer J..

[33] Han S, Tan S, Wang A (2022). Bacterial rather than fungal diversity and community assembly drive soil multifunctionality in a subtropical forest ecosystem. Env. Microbiol. Rep..

[34] Lynch MDJ, Neufeld JD (2015). Ecology and exploration of the rare biosphere. Nat. Rev. Microbiol..

[35] Pedrós-Alió C (2006). Marine microbial diversity: Can it be determined?. Trends Microbiol..

[36] Logares R, Mangot JF, Massana R (2015). Rarity in aquatic microbes: placing protists on the map. Res. Microbiol..

[37] Louca S, Polz MF, Mazel F, Albright MBN, Huber JA, O’Connor MI, Ackermann M, Hahn AS, Srivastava DS, Crowe SA, Doebeli M, Parfrey LW (2018). Function and functional redundancy in microbial systems. Nat. Ecol. Evol..

[38] Jousset A, Bienhold C, Chatzinotas A, Gallien L, Gobet A, Kurm V, Küsel K, Rilling MC, Rivett DW, Salles JF, van der Heijden MA, Youssef NH, Zhang X, Wei Z, Gera Hol WH (2017). Where less may be more: how the rare biosphere pulls ecosystems strings. ISME J..

[39] Dai TJ, Zhang Y, Tang YS, Bai YH, Tao YL, Huang B, Wen DH (2016). Identifying the key taxonomic categories that characterize microbial community diversity using full-scale classification: A case study of microbial communities in the sediments of Hangzhou Bay. FEMS Microbiol. Ecol..

[40] Wilson B, Hayek LAC (2015). Distinguishing relative specialist and generalist species in the fossil record. Mar. Micropa..

[41] Wu WX, Logares R, Huang BQ, Hsieh CH (2017). Abundant and rare picoeukaryotic sub-communities present contrasting patterns in the epipelagic waters of marginal seas in the northwestern Pacific Ocean. Environ. Microbiol..

[42] SD (2000). Soil Analysis in Agricultural Chemistry.

[43] Ministry of Environmental Protection, PRC. Soil-Determination of Dry Matter and Water Content-Gravimetric Method; HJ613-2011; Ministry of Environmental Protection, PRC: Beijing, China (2011) (**in Chinese**).

[44] Ministry of Environmental Protection, PRC. Soil Determination of Organic Carbon-Potassium Dichromate Oxidation Spectrophotometric Method; HJ615-2011; Ministry of Environmental Protection, PRC: Beijing, China (2011) (**in Chinese**).

[45] Ministry of Agriculture, PRC. Soil Testing-Method for Determination of Available Phosphorus in Soil; NY/T 1121.7-2014; Ministry of Agriculture, PRC: Beijing, China (2012) (**in Chinese**).

[46] Ministry of Environmental Protection, PRC. Soil-Determination of Ammonium, Nitrite and Nitrate by Extraction with Potassium Chloride Solution-Spectrophotometric Methods. HJ634-2012 Beijing, China (2012) (**in Chinese**).

[47] Standardization Administration of China. Determination of Nitrate Nitrogen in Soil-Ultraviolet Spectrophotometry Method. GB/T 32737-2016 Beijing, China (2016) (**in Chinese**).

[48] Bolger AM, Lohse M, Usadel (2014). Trimmomatic: A flexible trimmer for Illumina sequence data. Bioinformatics.

[49] Martin M (2011). Cut adapt removes adapter sequences from high-throughput sequencing reads. Embnet. J..

[50] Edgar RC (2013). UPARSE: Highly accurate OTU sequences from microbial amplicon reads. Nat. Methods.

[51] Edgar RC, Haas BJ, Clemente JC, Quince C, Knight R (2011). UCHIME improves sensitivity and speed of chimera detection. Bioinformatics.

[52] Wu JP, Liu WF, Zhang WX, Shao YH, Duan HL, Chen BD, Wei XH, Fan HB (2019). Long-term nitrogen addition changes soil microbial community and litter decomposition rate in a subtropical forest. Appl. Soil Ecol..

[53] Jiao S, Chen WM, Wei GH (2017). Biogeography and ecological diversity patterns of rare and abundant bacteria in oil-contaminated soils. Mol. Ecol..

[54] Xue YY, Chen HH, Yang RJ, Liu M, Huang BQ, Yang J (2018). Distinct patterns and processes of abundant and rare eukaryotic plankton communities following a reservoir cyanobacterial bloom. ISME J..

[55] Chen WD, Ren KX, Isabwe A, Chen HH, Liu M, Yang J (2019). Stochastic processes shape microeukaryotic community assembly in a sub-tropical river across wet and dry seasons. Microbiome.

[56] Levins R (1968). Evolution of diversity, efficiency and community stability. Am. Zool..

[57] Estavillo C, Pardini R, da Rocha PL (2013). Forest loss and the biodiversity threshold: An evaluation considering species habitat requirements and the use of matrix habitats. PLoS One.

[58] Wu C, Kan J, Narale DD (2022). Dynamics of bacterial communities during a seasonal hypoxia at the Bohai Sea: Coupling and response between abundant and rare populations. J. Environ. Sci..

[59] Buee M, Courty PE, Mignot D, Garbaye J (2007). Soil niche effect on species diversity and catabolic activities in an ectomycorrhizal fungal community. Soil Biol. Biochem..

[60] van der Voort M, Kempenaar M, van Driel M, Raaijmakers JM, Mendes R (2016). Impact of soil heat on reassembly of bacterial communities in the rhizosphere microbiome and plant disease suppression. Ecol. Lett..

[61] Xia ZC, Kong CH, Wang P, Chen LC, Wang SL (2012). Characteristics of soil microbial community structure in *Cunninghamia lanceolata* plantation. J. Appl. Ecol..

[62] Liao JQ, Cao XF, Zhao L, Wang J, Gao Z, Wang MC, Huang Y (2016). The importance of neutral and niche processes for bacterial community assembly differs between habitat generalists and specialists. FEMS Microbiol. Ecol..

[63] Lindh MV, Sjostedt J, Casini M, Andersson A, Legrand C, Pinhassi J (2016). Local environmental conditions shape generalist but not specialist components of microbial metacommunities in the Baltic Sea. Front. Microbiol..

[64] Drury WH (1974). Rare species. Biol. Conserv..

[65] Sogin ML, Morrison HG, Huber JA, Welch DM, Huse SM, Neal PR, Arrieta JM, Herndl GJ (2006). Microbial diversity in the deep sea and the underexplored “rare biosphere”. Proc. Natl. Acad. Sci. U. S. A..

[66] Kang P, Pan Y, Yang P, Hu J, Zhao T, Zhang Y, Ding X, Yan X (2022). A comparison of microbial composition under three tree ecosystems using the stochastic process and network complexity approaches. Front. Microbiol..

[67] Hearn AJ, Cushman SA, Ross J, Goossens B, Hunter LTB, Macdonald DW (2018). Spatio-temporal ecology of sympatric felids on Borneo. Evidence for resource partitioning?. PLoS One.

[68] Li PF, Li WT, Dumbrell AJ, Liu M, Li GL, Wu M, Jiang CY, Li ZP (2020). Spatial variation in soil fungal communities across paddy fields in subtropical China. mSystems.

