# Supplementary material for: Response of soil bacteria on habitat-specialization and abundance gradient to different afforestation types
Source: Sci Rep. 2023 Oct 24;13:18181. doi: 10.1038/s41598-023-44468-x (PMC10598043; doi:10.1038/s41598-023-44468-x)
Supplement: Supplementary file 1 — Supplementary Information. [file 41598_2023_44468_MOESM1_ESM.docx]

**Table S1：ANOVA and Monte Carlo analysis of effects of tree species composition on soil microbial structure of each habitat-specialization subgroup (TG：Habitat-generalized subgroup of topsoil; TN: Habitat-neutral subgroup of topsoil; TS: Habitat-specialized subgroup of topsoil; SG: Habitat-generalized subgroup of subsoil; SN: Habitat-neutral subgroup of subsoil; SS: Habitat-specialized subgroup of subsoil）**

|  | TG | | TN | | TS | | SG | | SN | | SS | |
| --- | --- | --- | --- | --- | --- | --- | --- | --- | --- | --- | --- | --- |
| RDA | F | P | F | P | F | P | F | P | F | P | F | P |
| (df=5) | 1.297 | **0.009** | 1.480 | **0.002** | 1.418 | **0.027** | 1.178 | 0.069 | 1.379 | **0.010** | 1.535 | **0.017** |
| Factors | r^2^ | P | r^2^ | P | r^2^ | P | r^2^ | P | r^2^ | P | r^2^ | P |
| X1 | 0.027 | 0.793 | 0.024 | 0.845 | 0.055 | 0.644 | 0.022 | 0.862 | 0.099 | 0.418 | 0.057 | 0.602 |
| X2 | 0.265 | 0.097 | 0.226 | 0.118 | 0.197 | 0.154 | 0.230 | 0.103 | 0.169 | 0.205 | 0.192 | 0.142 |
| X3 | 0.090 | 0.483 | 0.121 | 0.442 | 0.056 | 0.598 | 0.142 | 0.203 | 0.340 | 0.053 | 0.620 | 0.052 |
| X4 | 0.245 | 0.079 | 0.234 | 0.093 | 0.220 | 0.112 | 0.452 | **0.009** | 0.394 | **0.013** | 0.426 | **0.015** |
| X5 | 0.041 | 0.685 | 0.061 | 0.632 | 0.111 | 0.353 | 0.378 | **0.004** | 0.390 | **0.031** | 0.665 | **0.008** |
| X6 | 0.090 | 0.483 | 0.121 | 0.442 | 0.056 | 0.598 | 0.142 | 0.203 | 0.340 | 0.053 | 0.620 | 0.052 |
| X7 | 0.256 | 0.067 | 0.287 | 0.051 | 0.305 | 0.046 | 0.269 | 0.068 | 0.342 | **0.027** | 0.202 | 0.128 |
| X8 | 0.089 | 0.466 | 0.092 | 0.464 | 0.066 | 0.587 | 0.128 | 0.317 | 0.106 | 0.399 | 0.071 | 0.534 |
| X9 | 0.109 | 0.343 | 0.143 | 0.294 | 0.109 | 0.401 | 0.066 | 0.730 | 0.063 | 0.711 | 0.039 | 0.562 |
| X10 | 0.109 | 0.343 | 0.143 | 0.294 | 0.109 | 0.401 | 0.066 | 0.730 | 0.063 | 0.711 | 0.039 | 0.562 |
| X11 | 0.160 | 0.233 | 0.264 | 0.063 | 0.255 | 0.068 | 0.019 | 0.872 | 0.090 | 0.481 | 0.069 | 0.478 |
| X12 | 0.056 | 0.748 | 0.139 | 0.361 | 0.150 | 0.352 | 0.134 | 0.327 | 0.057 | 0.774 | 0.032 | 0.740 |
| X13 | 0.102 | 0.408 | 0.195 | 0.083 | 0.184 | 0.190 | 0.182 | 0.100 | 0.089 | 0.474 | 0.047 | 0.497 |
| X14 | 0.243 | 0.096 | 0.179 | 0.102 | 0.065 | 0.561 | 0.128 | 0.363 | 0.121 | 0.297 | 0.049 | 0.415 |
| X15 | 0.156 | 0.274 | 0.118 | 0.521 | 0.037 | 0.783 | 0.042 | 0.845 | 0.052 | 0.820 | 0.046 | 0.501 |
| X16 | 0.156 | 0.274 | 0.118 | 0.521 | 0.037 | 0.783 | 0.042 | 0.845 | 0.052 | 0.820 | 0.046 | 0.501 |


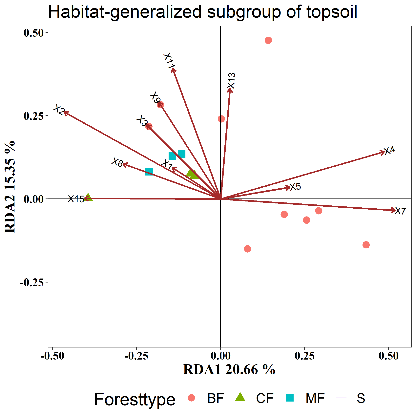

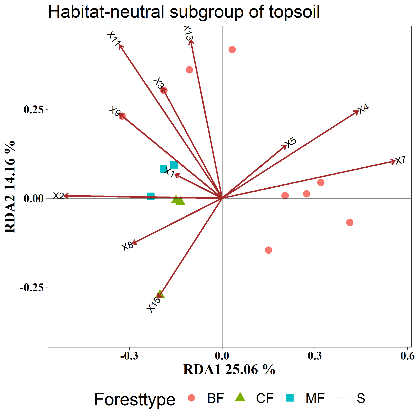

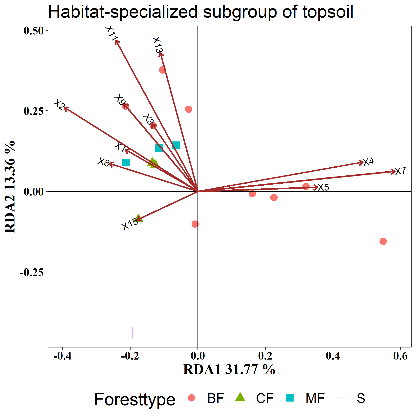

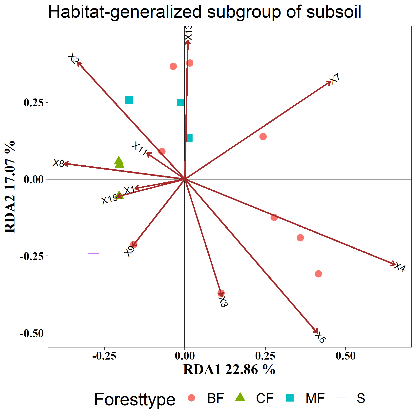

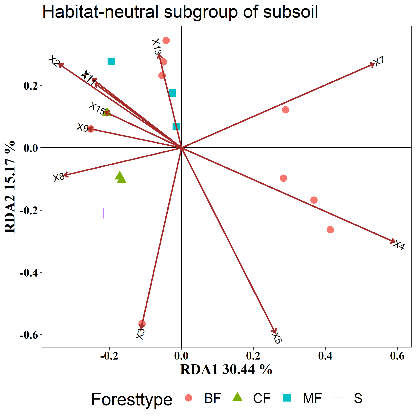

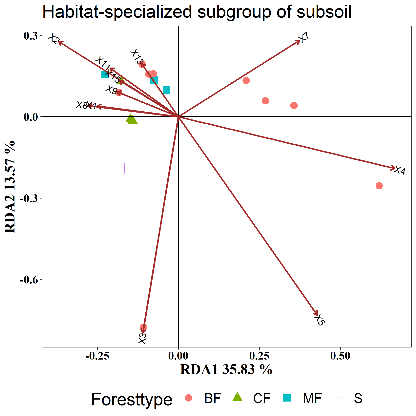


**Fig S1: RDA analysis of tree species composition on community structure of each habitat specialization subgroup (X1: *Quercus acutissima*; X2: *Pinus densiflora*; X3: *Platycladus orientalis*; X4: *Robinia pseudoacacia*; X5: *Morus alba*; X6: *Ziziphus jujuba* var. *spinosa*; X7: *Populus davidiana*; X8: *Pinus tabulaeformis*; X9: *Salix matsudana*; X10: *Zanthoxylum bungeanum*; X11: *Diospyros lotus*; X12: *Pterocarya stenoptera*; X13: *Prunus persica*; X14: *Koelreuteria paniculate*; X15: *Ailanthus altissima*; X16: *Ulmus pumila***

**Table S2：ANOVA and Monte Carlo analysis of effects of shrub species composition on soil microbial structure of each habitat-specialization subgroup (TG：Habitat-generalized subgroup of topsoil; TN: Habitat-neutral subgroup of topsoil; TS: Habitat-specialized subgroup of topsoil; SG: Habitat-generalized subgroup of subsoil; SN: Habitat-neutral subgroup of subsoil; SS: Habitat-specialized subgroup of subsoil）**

|  | TG | | TN | | TS | | SG | | SN | | SS | |
| --- | --- | --- | --- | --- | --- | --- | --- | --- | --- | --- | --- | --- |
| RDA | F | P | F | P | F | P | F | P | F | P | F | P |
| (df=5) | 1.085 | 0.244 | 1.211 | 0.055 | 1.331 | 0.097 | 1.245 | **0.037** | 1.312 | **0.021** | 1.393 | 0.118 |
| Factors | r^2^ | P | r^2^ | P | r^2^ | P | r^2^ | P | r^2^ | P | r^2^ | P |
| X1 | 0.233 | 0.099 | 0.365 | 0.029 | 0.343 | **0.032** | 0.306 | **0.048** | 0.246 | 0.081 | 0.210 | 0.120 |
| X2 | 0.037 | 0.739 | 0.039 | 0.805 | 0.025 | 0.825 | 0.040 | 0.772 | 0.077 | 0.561 | 0.057 | 0.537 |
| X3 | 0.017 | 0.944 | 0.011 | 1.000 | 0.014 | 0.905 | 0.054 | 0.787 | 0.102 | 0.301 | 0.030 | 0.852 |
| X4 | 0.009 | 0.938 | 0.066 | 0.558 | 0.035 | 0.726 | 0.115 | 0.412 | 0.138 | 0.317 | 0.201 | 0.146 |
| X5 | 0.296 | 0.052 | 0.233 | 0.086 | 0.284 | 0.066 | 0.443 | **0.008** | 0.456 | **0.009** | 0.599 | **0.009** |
| X6 | 0.128 | 0.369 | 0.138 | 0.308 | 0.056 | 0.529 | 0.079 | 0.617 | 0.344 | **0.050** | 0.652 | 0.054 |
| X7 | 0.084 | 0.502 | 0.072 | 0.628 | 0.052 | 0.638 | 0.104 | 0.418 | 0.409 | **0.007** | 0.702 | **0.005** |
| X8 | 0.152 | 0.242 | 0.119 | 0.427 | 0.052 | 0.573 | 0.055 | 0.784 | 0.052 | 0.754 | 0.045 | 0.486 |
| X9 | 0.134 | 0.279 | 0.157 | 0.203 | 0.222 | 0.100 | 0.165 | 0.169 | 0.229 | 0.093 | 0.154 | 0.186 |
| X10 | 0.094 | 0.437 | 0.126 | 0.414 | 0.212 | 0.200 | 0.137 | 0.298 | 0.196 | 0.141 | 0.159 | 0.211 |
| X11 | 0.164 | 0.158 | 0.169 | 0.156 | 0.205 | 0.221 | 0.087 | 0.489 | 0.107 | 0.289 | 0.115 | 0.219 |
| X12 | 0.180 | 0.169 | 0.098 | 0.464 | 0.047 | 0.709 | 0.119 | 0.320 | 0.179 | 0.191 | 0.079 | 0.400 |
| X13 | 0.412 | 0.057 | 0.280 | **0.038** | 0.129 | 0.288 | 0.105 | 0.335 | 0.100 | 0.350 | 0.051 | 0.438 |
| X14 | 0.412 | 0.057 | 0.280 | **0.038** | 0.129 | 0.288 | 0.105 | 0.335 | 0.100 | 0.350 | 0.051 | 0.438 |
| X15 | 0.412 | 0.057 | 0.280 | **0.038** | 0.129 | 0.288 | 0.105 | 0.335 | 0.100 | 0.350 | 0.051 | 0.438 |
| X16 | 0.186 | 0.145 | 0.181 | 0.157 | 0.219 | 0.167 | 0.088 | 0.484 | 0.066 | 0.596 | 0.032 | 0.703 |
| X17 | 0.059 | 0.689 | 0.120 | 0.472 | 0.115 | 0.395 | 0.102 | 0.438 | 0.070 | 0.565 | 0.038 | 0.560 |
| X18 | 0.059 | 0.689 | 0.120 | 0.472 | 0.115 | 0.395 | 0.102 | 0.438 | 0.070 | 0.565 | 0.038 | 0.560 |


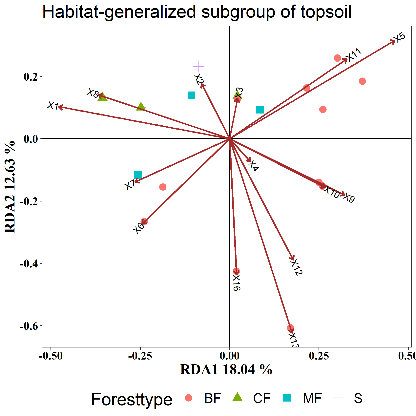

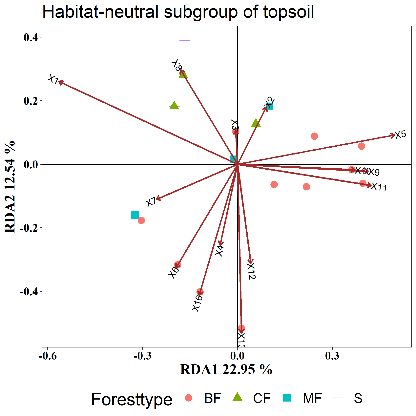

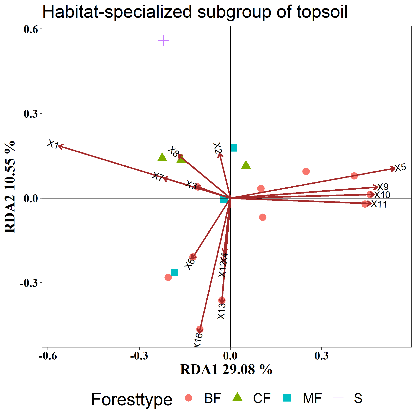

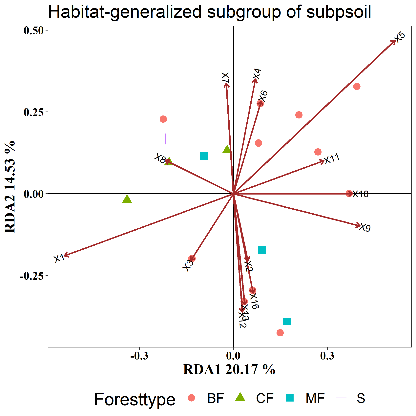

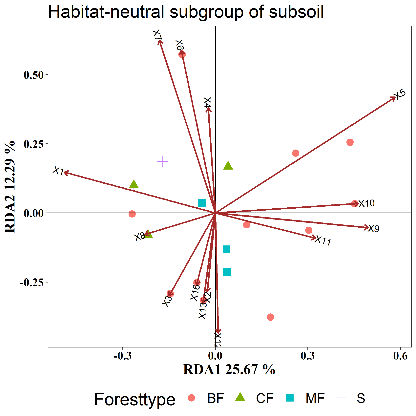

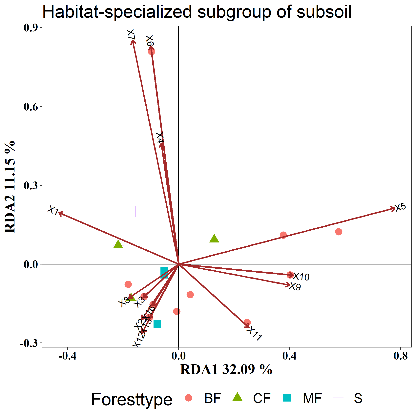


**Fig S2: RDA analysis of shrub species composition on community structure of each habitat specialization subgroup (X1: *Vitex negundo* var.; X2: *Quercus acutissima*; X3: *Morus alba*; X4: *Grewia biloba* var. *parviflora*; X5: *Robinia pseudoacacia*; X6: *Morus mongolica*; X7: *Ziziphus jujuba* var. *spinosa*; X8: *Ailanthus altissima*; X9: *Amorpha fruticose*; X10: *Pinus tabulaeformis*; X11: *Diospyros lotus*; X12: *Forsythia suspensa*; X13: *Koelreuteria paniculate*; X14:** ***Leptopus chinensis*; X15: *Prunus persica*; X16: *Vitis amurensis*; X17: *Deutzia grandiflora*; X18: *Rubus crataegifolius*)**

**Table S3：ANOVA and Monte Carlo analysis of effects of tree species composition on soil microbial structure of each abundance subgroup (TA: Abundant subgroup of topsoil; TM: Medium abundance subgroup of topsoil; TR: Rare subgroup of topsoil; SA: Abundance subgroup of subsoil; SM: Medium abundance subgroup of subsoil; SR: Rare subgroup of subsoil)**

|  | TA | | TM | | TR | | SA | | SM | | SR | |
| --- | --- | --- | --- | --- | --- | --- | --- | --- | --- | --- | --- | --- |
| RDA | F | P | F | P | F | P | F | P | F | P | F | P |
| (df=5) | 1.334 | 0.062 | 1.605 | **0.004** | 1.49 | **0.005** | 1.696 | **0.025** | 1.318 | 0.096 | 1.529 | **0.010** |
| Factors | r^2^ | P | r^2^ | P | r^2^ | P | r^2^ | P | r^2^ | P | r^2^ | P |
| X1 | 0.368 | **0.015** | 0.032 | 0.805 | 0.047 | 0.677 | 0.137 | 0.256 | 0.031 | 0.793 | 0.057 | 0.622 |
| X2 | 0.328 | **0.046** | 0.188 | 0.175 | 0.177 | 0.173 | 0.258 | 0.067 | 0.191 | 0.175 | 0.191 | 0.146 |
| X3 | 0.010 | 0.953 | 0.039 | 0.904 | 0.056 | 0.592 | 0.681 | **0.042** | 0.270 | 0.106 | 0.620 | **0.050** |
| X4 | 0.216 | 0.125 | 0.230 | 0.096 | 0.255 | **0.072** | 0.456 | **0.012** | 0.492 | **0.003** | 0.477 | **0.012** |
| X5 | 0.062 | 0.610 | 0.060 | 0.643 | 0.148 | 0.251 | 0.456 | **0.030** | 0.227 | **0.088** | 0.726 | **0.005** |
| X6 | 0.010 | 0.953 | 0.039 | 0.904 | 0.056 | 0.592 | 0.681 | **0.042** | 0.270 | 0.106 | 0.620 | **0.050** |
| X7 | 0.207 | 0.131 | 0.297 | **0.040** | 0.263 | **0.052** | 0.134 | 0.310 | 0.127 | 0.309 | 0.218 | 0.117 |
| X8 | 0.188 | 0.180 | 0.094 | 0.460 | 0.070 | 0.599 | 0.136 | 0.295 | 0.193 | 0.145 | 0.085 | 0.424 |
| X9 | 0.105 | 0.511 | 0.214 | 0.144 | 0.111 | 0.467 | 0.095 | 0.334 | 0.104 | 0.508 | 0.037 | 0.718 |
| X10 | 0.105 | 0.511 | 0.214 | 0.144 | 0.111 | 0.467 | 0.095 | 0.334 | 0.104 | 0.508 | 0.037 | 0.718 |
| X11 | 0.183 | 0.237 | 0.491 | **0.003** | 0.252 | 0.082 | 0.073 | 0.468 | 0.085 | 0.503 | 0.060 | 0.604 |
| X12 | 0.143 | 0.246 | 0.263 | 0.096 | 0.149 | 0.254 | 0.003 | 0.959 | 0.003 | 1.000 | 0.029 | 0.907 |
| X13 | 0.180 | 0.085 | 0.280 | 0.036 | 0.198 | 0.151 | 0.004 | 0.950 | 0.008 | 0.922 | 0.042 | 0.684 |
| X14 | 0.085 | 0.647 | 0.045 | 0.848 | 0.127 | 0.392 | 0.079 | 0.382 | 0.046 | 0.625 | 0.041 | 0.516 |
| X15 | 0.099 | 0.562 | 0.268 | **0.061** | 0.049 | 0.660 | 0.121 | 0.297 | 0.228 | 0.206 | 0.049 | 0.421 |
| X16 | 0.099 | 0.562 | 0.268 | **0.061** | 0.049 | 0.660 | 0.121 | 0.297 | 0.228 | 0.206 | 0.049 | 0.421 |


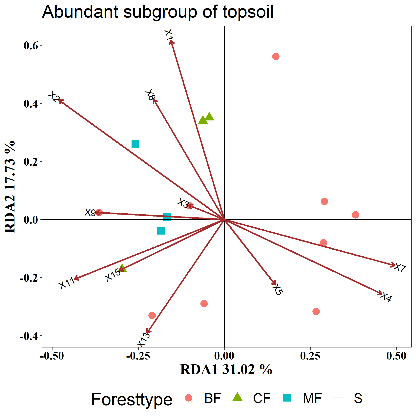

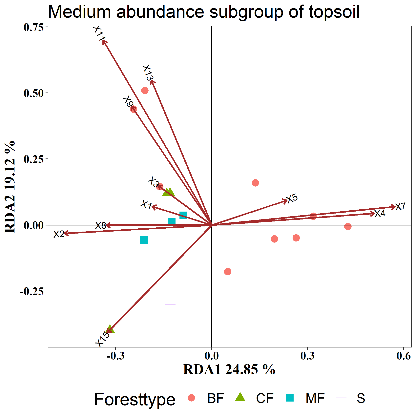

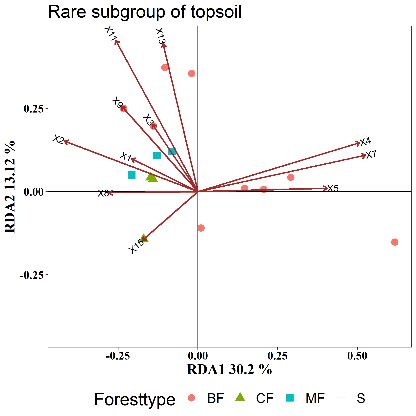

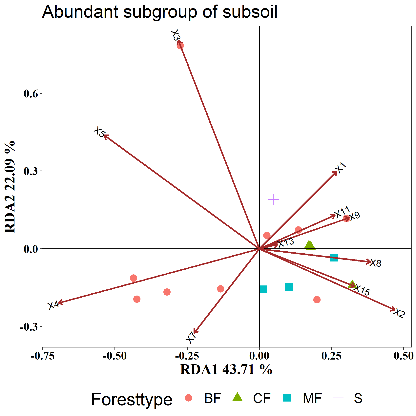

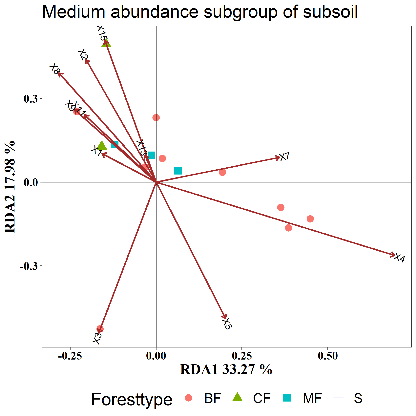

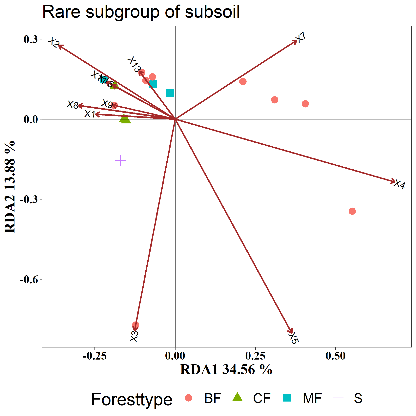


**Fig S3: RDA analysis of tree species composition on community structure of each abundance subgroup (X1: *Quercus acutissima*; X2: *Pinus densiflora*; X3: *Platycladus orientalis*; X4: *Robinia pseudoacacia*; X5: *Morus alba*; X6: *Ziziphus jujuba* var. *spinosa*; X7: *Populus davidiana*; X8: *Pinus tabulaeformis*; X9: *Salix matsudana*; X10: *Zanthoxylum bungeanum*; X11: *Diospyros lotus*; X12: *Pterocarya stenoptera*; X13: *Prunus persica*; X14: *Koelreuteria paniculate*; X15: *Ailanthus altissima*; X16: *Ulmus pumila*)**

**Table S4：ANOVA and Monte Carlo analysis of effects of shrub species composition on soil microbial structure of each abundance subgroup (TA: Abundant subgroup of topsoil; TM: Medium abundance subgroup of topsoil; TR: Rare subgroup of topsoil; SA: Abundance subgroup of subsoil; SM: Medium abundance subgroup of subsoil; SR: Rare subgroup of subsoil)**

|  | TA | | TM | | TR | | SA | | SM | | SR | |
| --- | --- | --- | --- | --- | --- | --- | --- | --- | --- | --- | --- | --- |
| RDA | F | P | F | P | F | P | F | P | F | P | F | P |
| (df=5) | 0.815 | 0.898 | 1.109 | 0.299 | 1.292 | 0.105 | 1.101 | 0.393 | 1.162 | 0.291 | 1.343 | 0.074 |
| Factors | r^2^ | P | r^2^ | P | r^2^ | P | r^2^ | P | r^2^ | P | r^2^ | P |
| X1 | 0.220 | 0.125 | 0.340 | **0.031** | 0.361 | **0.021** | 0.177 | 0.179 | 0.314 | **0.032** | 0.215 | 0.129 |
| X2 | 0.207 | 0.097 | 0.093 | 0.469 | 0.021 | 0.855 | 0.044 | 0.655 | 0.003 | 0.964 | 0.045 | 0.689 |
| X3 | 0.435 | 0.053 | 0.016 | 0.957 | 0.011 | 0.953 | 0.064 | 0.599 | 0.007 | 0.910 | 0.033 | 0.862 |
| X4 | 0.003 | 0.984 | 0.009 | 0.917 | 0.037 | 0.770 | 0.173 | 0.224 | 0.044 | 0.663 | 0.131 | 0.225 |
| X5 | 0.182 | 0.173 | 0.255 | 0.057 | 0.334 | **0.042** | 0.392 | 0.029 | 0.369 | 0.030 | 0.588 | 0.006 |
| X6 | 0.010 | 1.000 | 0.035 | 0.893 | 0.060 | 0.555 | 0.610 | 0.070 | 0.083 | 0.554 | 0.622 | 0.061 |
| X7 | 0.019 | 0.914 | 0.046 | 0.679 | 0.050 | 0.641 | 0.589 | **0.016** | 0.388 | 0.057 | 0.643 | **0.008** |
| X8 | 0.104 | 0.413 | 0.276 | 0.051 | 0.064 | 0.444 | 0.124 | 0.263 | 0.161 | 0.177 | 0.048 | 0.447 |
| X9 | 0.101 | 0.419 | 0.140 | 0.224 | 0.188 | 0.198 | 0.089 | 0.442 | 0.136 | 0.257 | 0.145 | 0.190 |
| X10 | 0.077 | 0.633 | 0.106 | 0.338 | 0.177 | 0.261 | 0.076 | 0.549 | 0.121 | 0.368 | 0.144 | 0.204 |
| X11 | 0.171 | 0.065 | 0.173 | 0.170 | 0.172 | 0.220 | 0.116 | 0.297 | 0.063 | 0.594 | 0.146 | 0.185 |
| X12 | 0.002 | 0.984 | 0.089 | 0.480 | 0.073 | 0.530 | 0.026 | 0.807 | 0.008 | 0.945 | 0.078 | 0.461 |
| X13 | 0.126 | 0.268 | 0.059 | 0.686 | 0.223 | 0.165 | 0.086 | 0.488 | 0.028 | 0.622 | 0.042 | 0.601 |
| X14 | 0.126 | 0.268 | 0.059 | 0.686 | 0.223 | 0.165 | 0.086 | 0.488 | 0.028 | 0.622 | 0.042 | 0.601 |
| X15 | 0.126 | 0.268 | 0.059 | 0.686 | 0.223 | 0.165 | 0.086 | 0.488 | 0.028 | 0.622 | 0.042 | 0.601 |
| X16 | 0.159 | 0.098 | 0.319 | 0.051 | 0.211 | 0.182 | 0.008 | 0.896 | 0.012 | 0.811 | 0.031 | 0.905 |
| X17 | 0.123 | 0.285 | 0.235 | 0.159 | 0.109 | 0.348 | 0.101 | 0.463 | 0.130 | 0.325 | 0.036 | 0.704 |
| X18 | 0.123 | 0.285 | 0.235 | 0.159 | 0.109 | 0.348 | 0.101 | 0.463 | 0.130 | 0.325 | 0.036 | 0.704 |


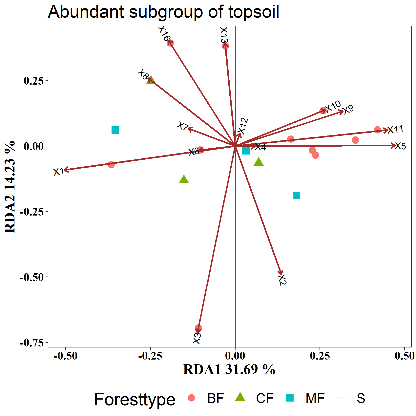

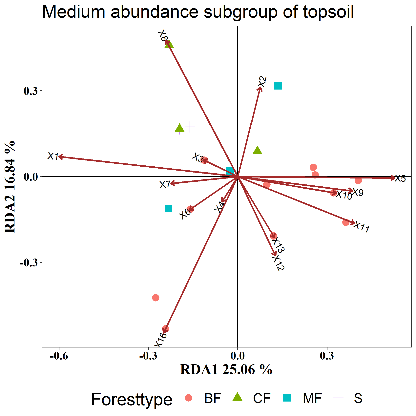

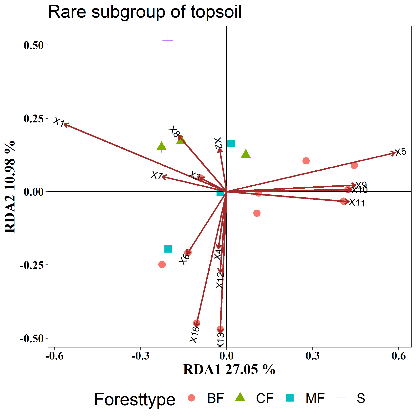

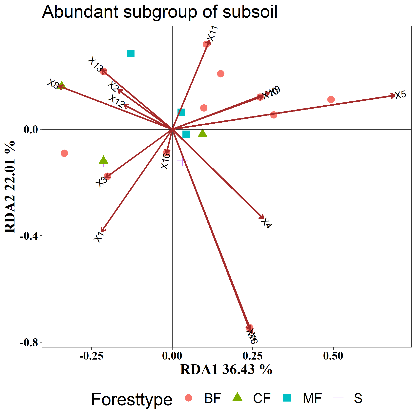

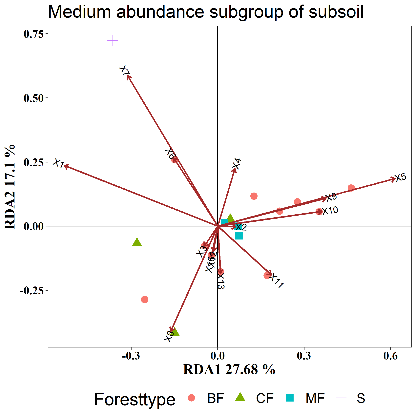

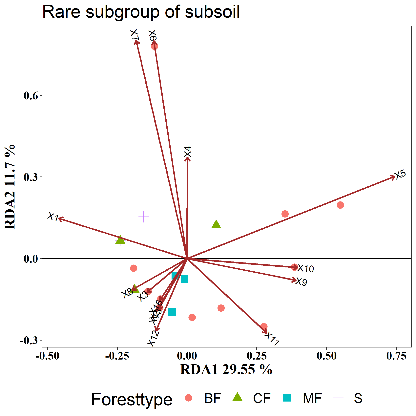


**Fig S4: RDA analysis of shrub species composition on community structure of each habitat abundance subgroup (X1: *Vitex negundo* var.; X2: *Quercus acutissima*; X3: *Morus alba*; X4: *Grewia biloba* var. *parviflora*; X5: *Robinia pseudoacacia*; X6: *Morus mongolica*; X7: *Ziziphus jujuba* var. *spinosa*; X8: *Ailanthus altissima*; X9: *Amorpha fruticose*; X10: *Pinus tabulaeformis*; X11: *Diospyros lotus*; X12: *Forsythia suspensa*; X13: *Koelreuteria paniculate*; X14:** ***Leptopus chinensis*; X15: *Prunus persica*; X16: *Vitis amurensis*; X17: *Deutzia grandiflora*; X18: *Rubus crataegifolius*)**

**Table S5：ANOVA and Monte Carlo analysis of effects of vegetation diversity on soil microbial structure of each habitat-specialization subgroup (TG：Habitat-generalized subgroup of topsoil; TN: Habitat-neutral subgroup of topsoil; TS: Habitat-specialized subgroup of topsoil; SG: Habitat-generalized subgroup of subsoil; SN: Habitat-neutral subgroup of subsoil; SS: Habitat-specialized subgroup of subsoil）**

|  | TG | | TN | | TS | | SG | | SN | | SS | |
| --- | --- | --- | --- | --- | --- | --- | --- | --- | --- | --- | --- | --- |
| RDA | F | P | F | P | F | P | F | P | F | P | F | P |
| (df=5) | 1.38 | **0.003** | 1.524 | **0.004** | 1.729 | **0.002** | 1.337 | **0.012** | 1.580 | **0.003** | 1.724 | **0.004** |
| Factors | r^2^ | P | r^2^ | P | r^2^ | P | r^2^ | P | r^2^ | P | r^2^ | P |
| SHI | 0.797 | **0.001** | 0.707 | **0.001** | 0.657 | **0.001** | 0.105 | 0.401 | 0.253 | 0.087 | 0.256 | 0.079 |
| SII | 0.845 | **0.001** | 0.725 | **0.001** | 0.732 | **0.001** | 0.026 | 0.792 | 0.094 | 0.422 | 0.082 | 0.503 |
| SHE | 0.567 | **0.005** | 0.593 | **0.001** | 0.625 | **0.003** | 0.502 | **0.007** | 0.423 | **0.007** | 0.376 | **0.025** |
| SIE | 0.477 | **0.009** | 0.503 | **0.003** | 0.533 | **0.006** | 0.541 | **0.006** | 0.412 | **0.009** | 0.376 | **0.022** |
| PIE | 0.746 | **0.001** | 0.721 | **0.001** | 0.738 | **0.001** | 0.295 | 0.053 | 0.384 | **0.017** | 0.292 | **0.048** |


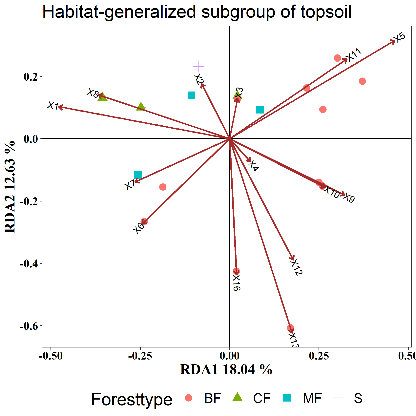

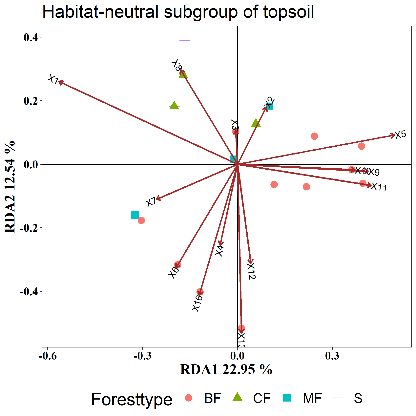

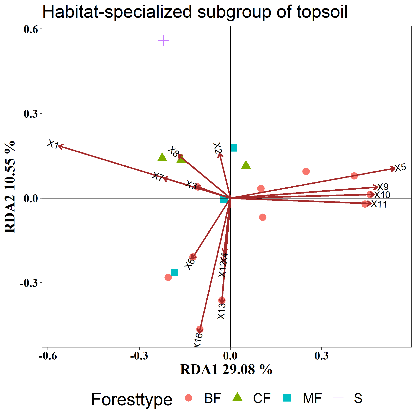

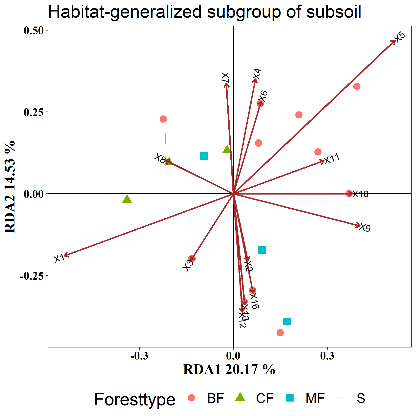

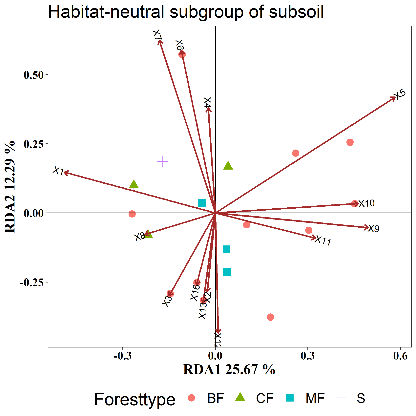

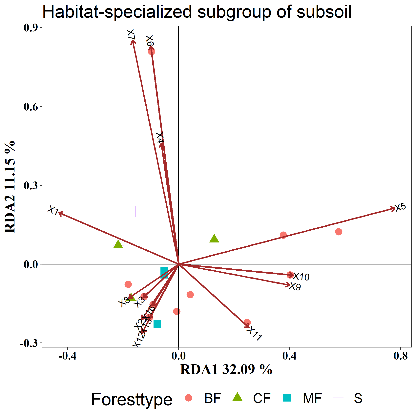


**Fig S5: RDA analysis of vegetation diversity on community structure of each habitat specialization subgroup (SHI: Shannon-winner diversity index; SII: Simpson diversity index; SHE: Shannon-winner evenness index; SIE: Simspon evenness index; PIE: Pielou evenness index)**

**Table S6：ANOVA and Monte Carlo analysis of effects of vegetation diversity on soil microbial structure of each abundance subgroup (TA: Abundant subgroup of topsoil; TM: Medium abundance subgroup of topsoil; TR: Rare subgroup of topsoil; SA: Abundance subgroup of subsoil; SM: Medium abundance subgroup of subsoil; SR: Rare subgroup of subsoil)**

|  | TA | | TM | | TR | | SA | | SM | | SR | |
| --- | --- | --- | --- | --- | --- | --- | --- | --- | --- | --- | --- | --- |
| RDA | F | P | F | P | F | P | F | P | F | P | F | P |
| (df=5) | 1.180 | 0.194 | 1.343 | **0.036** | 1.143 | 0.171 | 1.108 | 0.360 | 0.965 | 0.539 | 1.062 | 0.336 |
| Factors | r^2^ | P | r^2^ | P | r^2^ | P | r^2^ | P | r^2^ | P | r^2^ | P |
| SHI | 0.538 | **0.004** | 0.692 | **0.001** | 0.598 | **0.001** | 0.431 | **0.007** | 0.467 | **0.006** | 0.434 | **0.010** |
| SII | 0.540 | **0.004** | 0.719 | **0.001** | 0.590 | **0.001** | 0.439 | **0.010** | 0.490 | **0.003** | 0.468 | **0.005** |
| SHE | 0.176 | 0.187 | 0.161 | 0.220 | 0.546 | **0.002** | 0.089 | 0.432 | 0.350 | **0.020** | 0.079 | 0.491 |
| SIE | 0.190 | 0.170 | 0.221 | 0.126 | 0.458 | **0.006** | 0.071 | 0.526 | 0.294 | **0.044** | 0.036 | 0.719 |
| PIE | 0.675 | **0.001** | 0.581 | **0.001** | 0.603 | **0.001** | 0.356 | **0.022** | 0.386 | **0.012** | 0.453 | **0.002** |


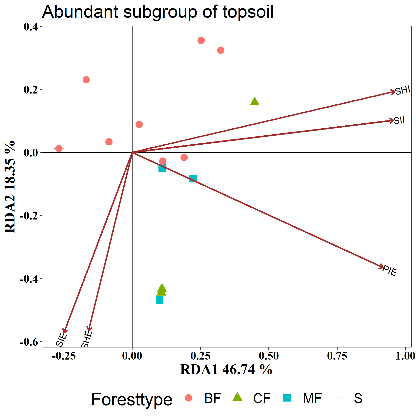

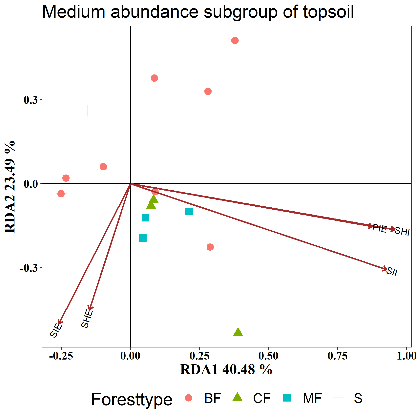

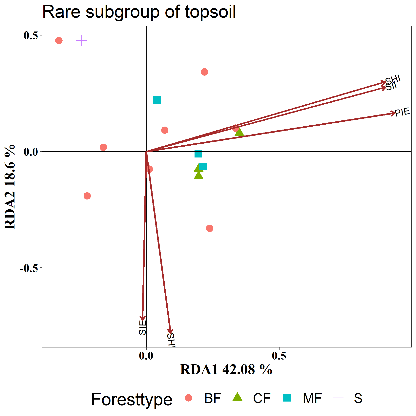

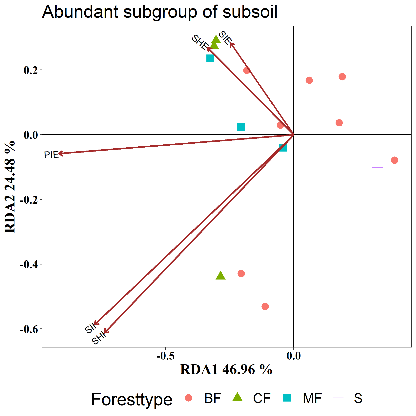

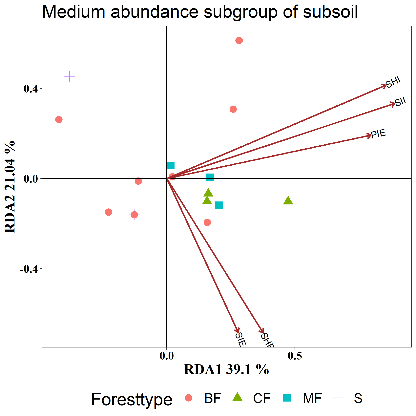

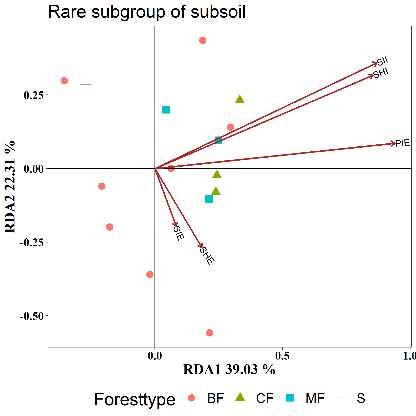


**Fig S6: RDA analysis of vegetation diversity on community structure of each abundance subgroup (SHI: Shannon-winner diversity index; SII: Simpson diversity index; SHE: Shannon-winner evenness index; SIE: Simspon evenness index; PIE: Pielou evenness index)**

**Table S7：ANOVA and Monte Carlo analysis of effects of soil chemical properties on soil microbial structure of each habitat-specialization subgroup (TG：Habitat-generalized subgroup of topsoil; TN: Habitat-neutral subgroup of topsoil; TS: Habitat-specialized subgroup of topsoil; SG: Habitat-generalized subgroup of subsoil; SN: Habitat-neutral subgroup of subsoil; SS: Habitat-specialized subgroup of subsoil）**

|  | TG | | TN | | TS | | SG | | SN | | SS | |
| --- | --- | --- | --- | --- | --- | --- | --- | --- | --- | --- | --- | --- |
| RDA | F | P | F | P | F | P | F | P | F | P | F | P |
| (df=5) | 1.142 | 0.099 | 1.356 | **0.008** | 1.553 | **0.001** | 0.960 | **0.608** | 1.017 | 0.433 | 1.229 | 0.134 |
| Factors | r^2^ | P | r^2^ | P | r^2^ | P | r^2^ | P | r^2^ | P | r^2^ | P |
| DM | 0.442 | **0.008** | 0.050 | 0.653 | 0.105 | 0.349 | 0.128 | 0.313 | 0.161 | 0.202 | 0.801 | **0.001** |
| AP | 0.148 | 0.242 | 0.205 | 0.143 | 0.052 | 0.644 | 0.005 | 0.961 | 0.057 | 0.606 | 0.040 | 0.688 |
| SOC | 0.696 | **0.001** | 0.166 | 0.202 | 0.121 | 0.344 | 0.143 | 0.279 | 0.485 | **0.004** | 0.297 | **0.046** |
| AN | 0.288 | 0.057 | 0.470 | **0.002** | 0.289 | 0.084 | 0.240 | 0.108 | 0.370 | **0.025** | 0.032 | 0.721 |
| NAN | 0.111 | 0.365 | 0.102 | 0.410 | 0.203 | 0.158 | 0.397 | **0.010** | 0.184 | 0.148 | 0.497 | **0.017** |
| pH | 0.197 | 0.157 | 0.469 | **0.007** | 0.636 | **0.002** | 0.210 | 0.129 | 0.074 | 0.508 | 0.109 | 0.360 |


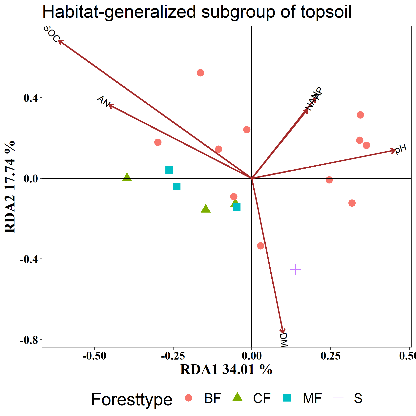

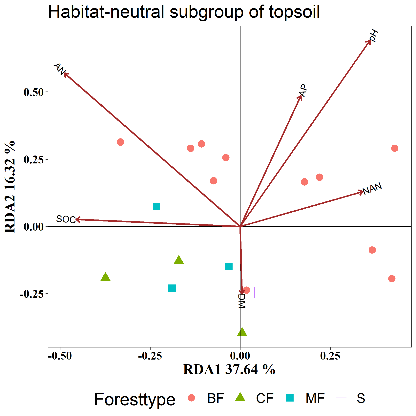

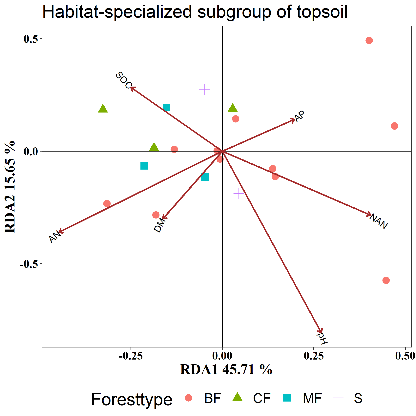

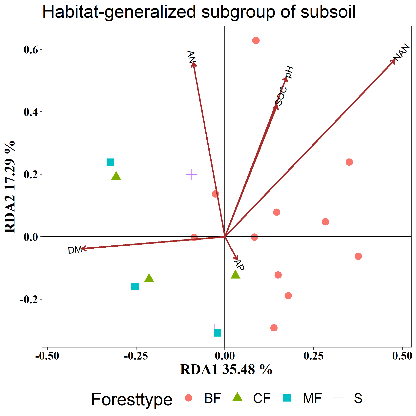

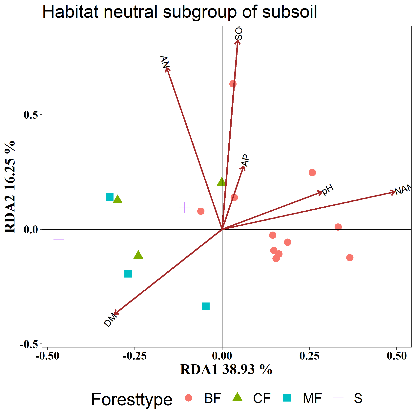

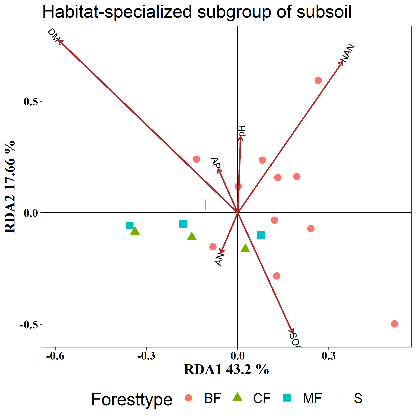


**Fig S7: RDA analysis of soil chemical properties on community structure of each habitat specialization subgroup (DM: Dry matter content; AP: Available phosphorus; SOC: Soil organic carbon; AN: Ammonium nitrogen; NAN: Nitrate nitrogen)**

**Table S8：ANOVA and Monte Carlo analysis of effects of soil chemical properties on soil microbial structure of each abundance subgroup (TA: Abundant subgroup of topsoil; TM: Medium abundance subgroup of topsoil; TR: Rare subgroup of topsoil; SA: Abundance subgroup of subsoil; SM: Medium abundance subgroup of subsoil; SR: Rare subgroup of subsoil)**

|  | TA | | TM | | TR | | SA | | SM | | SR | |
| --- | --- | --- | --- | --- | --- | --- | --- | --- | --- | --- | --- | --- |
| RDA | F | P | F | P | F | P | F | P | F | P | F | P |
|  | 1.274 | 0.095 | 1.223 | 0.099 | 1.525 | **0.003** | 1.091 | 0.348 | 0.951 | 0.563 | 1.203 | 0.134 |
| Factors | r^2^ | P | r^2^ | P | r^2^ | P | r^2^ | P | r^2^ | P | r^2^ | P |
| DM | 0.105 | 0.405 | 0.290 | 0.052 | 0.037 | 0.696 | 0.045 | 0.707 | 0.340 | **0.025** | 0.755 | **0.001** |
| AP | 0.454 | **0.002** | 0.065 | 0.573 | 0.047 | 0.671 | 0.004 | 0.963 | 0.196 | 0.152 | 0.061 | 0.581 |
| SOC | 0.230 | 0.110 | 0.167 | 0.196 | 0.077 | 0.506 | 0.185 | 0.186 | 0.224 | 0.119 | 0.301 | **0.039** |
| AN | 0.205 | 0.151 | 0.572 | **0.001** | 0.360 | **0.028** | 0.162 | 0.222 | 0.311 | **0.040** | 0.051 | 0.608 |
| NAN | 0.587 | **0.001** | 0.139 | 0.293 | 0.201 | 0.149 | 0.367 | **0.012** | 0.076 | 0.551 | 0.407 | **0.032** |
| pH | 0.150 | 0.260 | 0.326 | **0.024** | 0.620 | **0.001** | 0.148 | 0.259 | 0.021 | 0.841 | 0.293 | **0.048** |


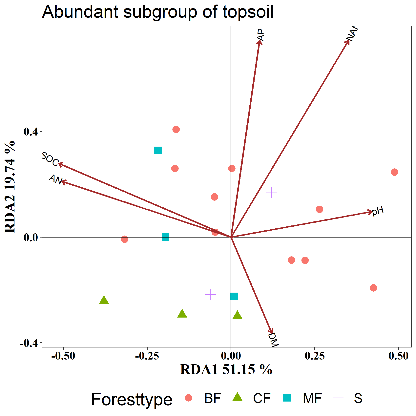

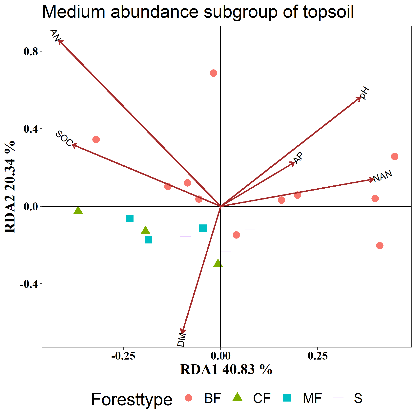

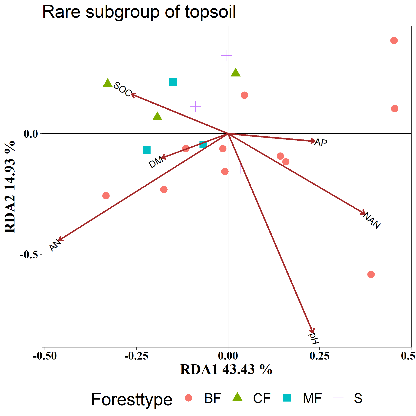

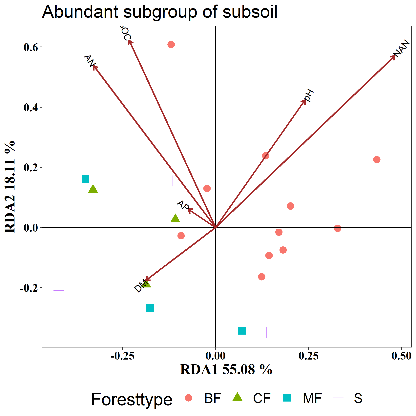

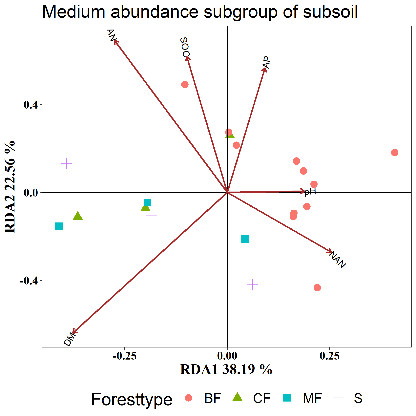

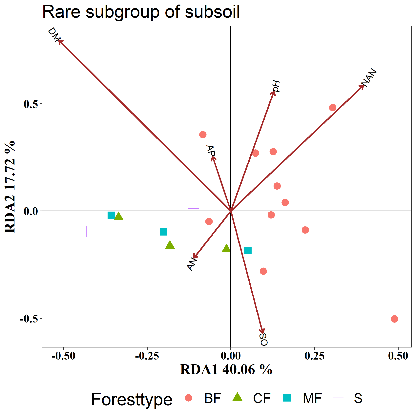


**Fig S8: RDA analysis of soil chemical properties on community structure of each abundance subgroup (DM: Dry matter content; AP: Available phosphorus; SOC: Soil organic carbon; AN: Ammonium nitrogen; NAN: Nitrate nitrogen)**
